# Regulation of Brain Tumor Dispersal by NKCC1 Through a Novel Role in Focal Adhesion Regulation

**DOI:** 10.1371/journal.pbio.1001320

**Published:** 2012-05-01

**Authors:** Tomas Garzon-Muvdi, Paula Schiapparelli, Colette ap Rhys, Hugo Guerrero-Cazares, Christopher Smith, Deok-Ho Kim, Lyonell Kone, Harrison Farber, Danielle Y. Lee, Steven S. An, Andre Levchenko, Alfredo Quiñones-Hinojosa

**Affiliations:** 1 Department of Neurosurgery, Johns Hopkins University School of Medicine, Baltimore, Maryland, United States of America; 2 Department of Biomedical Engineering, Johns Hopkins University School of Medicine, Baltimore, Maryland, United States of America; 3 Department of Bioengineering, University of Washington, Seattle, Washington, United States of America; 4 Department of Environmental Health Sciences, Johns Hopkins University School of Medicine, Baltimore, Maryland, United States of America; 5 Department of Physical Sciences in Oncology Center, Johns Hopkins University School of Medicine, Baltimore, Maryland, United States of America; 6 Department of Oncology, Johns Hopkins University School of Medicine, Baltimore, Maryland, United States of America; Cleveland Clinic, United States of America

## Abstract

Glioblastoma (GB) is a highly invasive and lethal brain tumor due to its universal recurrence. Although it has been suggested that the electroneutral Na^+^-K^+^-Cl^−^ cotransporter 1 (NKCC1) can play a role in glioma cell migration, the precise mechanism by which this ion transporter contributes to GB aggressiveness remains poorly understood. Here, we focused on the role of NKCC1 in the invasion of human primary glioma cells in vitro and in vivo. NKCC1 expression levels were significantly higher in GB and anaplastic astrocytoma tissues than in grade II glioma and normal cortex. Pharmacological inhibition and shRNA-mediated knockdown of NKCC1 expression led to decreased cell migration and invasion in vitro and in vivo. Surprisingly, knockdown of NKCC1 in glioma cells resulted in the formation of significantly larger focal adhesions and cell traction forces that were approximately 40% lower than control cells. Epidermal growth factor (EGF), which promotes migration of glioma cells, increased the phosphorylation of NKCC1 through a PI3K-dependant mechanism. This finding is potentially related to WNK kinases. Taken together, our findings suggest that NKCC1 modulates migration of glioma cells by two distinct mechanisms: (1) through the regulation of focal adhesion dynamics and cell contractility and (2) through regulation of cell volume through ion transport. Due to the ubiquitous expression of NKCC1 in mammalian tissues, its regulation by WNK kinases may serve as new therapeutic targets for GB aggressiveness and can be exploited by other highly invasive neoplasms.

## Abstract 2

The ion transporter NKCC1 determines brain tumor cell migration by regulating the interplay between cell adhesion and growth factor signaling, and is a potential therapeutic target to treat brain cancer.

## Author Summary

Treatment of many cancers has been hampered by the invasive ability of tumor cells. A notable example is brain cancer, which is incurable due to its invasiveness and resulting high tumor recurrence after surgical resection. Here, we analyze further the function of NKCC1, an ion transporter that is known to regulate cell volume and intracellular chloride concentration, and to play an important role in brain tumor cell invasion. Our findings suggest that in addition to its conventional function as an ion transporter, NKCC1 may also interact with the cytoskeleton and affect brain tumor cell migration by acting as an anchor that transduces contractile forces from the plasma membrane to the extracellular matrix en route to cell migration. Moreover, we show that regulation of NKCC1 by a family of unconventional enzymes, the WNK kinases, is an important factor that affects the activity of NKCC1 and may determine the invasive ability of brain tumor cells. We postulate that NKCC1 has multiple functions in brain tumor cell migration and that together with its regulatory enzymes may be therapeutic targets in the treatment of brain tumors or other types of cancer, given the wide expression of these proteins throughout the body.

## Introduction

Glioblastoma (GB) is the most common malignant primary brain tumor. GBs are aggressive and display key features of invasion and infiltration of healthy brain tissue [1]. Due to its invasive nature, GB is not curable through surgical resection [2],[3]. The surgical and medical treatment for patients with this disease has evolved in the last 20 years, however the prognosis remains dismal due to tumor recurrence [4]. Thus, understanding the mechanisms that GB cells utilize during migration and invasion into normal brain tissue is paramount in the development of novel, effective therapies.

Volume regulation, cytoskeletal rearrangements, and adhesion dynamics are major determinants of cell migration and are essential processes in invasion [5],[6]. Migration of mammalian cells is accompanied by volume changes. For instance, neutrophils [7] and dendritic cells [8] undergo cell volume increases when exposed to signals leading to migratory responses. Indeed, it has been hypothesized that inhibition of cell volume regulation impairs cell migration [9],[10]. NKCC1, a transporter that belongs to the SLC12A family of cation-chloride cotransporters, is a fundamental transporter utilized in the regulation of intracellular volume and in the accumulation of intracellular Cl^−^ [11],[12]. NKCC1 mediates the movement of Na^+^, K^+^, and Cl^−^ ions across the plasma membrane using the energy stored in the Na^+^ gradient, generated by the Na^+^/K^+^ ATPase. Recent work supports the notion that intracellular volume regulation by NKCC1 [13],[14], as well as aquaporin 4 (AQP4) [15], may indeed promote glioma cell invasion. However, whether cell volume regulation is the only or primary mechanism mediating NKCC1 effects is unclear. It is equally unclear if NKCC1 is differentially regulated in invasive cells.

In addition to cell volume regulation, ion transporters can participate in anchoring the cytoskeleton to the plasma membrane by binding to ezrin-radixin-moesin (ERM) proteins [16],[17]. ERM proteins associate directly with actin and integral membrane proteins, which connect the cytoskeleton to the plasma membrane [18]. Anion exchangers (AE) 1, 2, and 3, Na^+^/H^+^ exchanger 1 (NHE1), and a Na^+^/Ca^++^ exchanger are all able to act as cytoskeletal anchors by interacting with ERM proteins [19]. It has been shown that ERM proteins bind to clusters of positive amino acids in the juxtamembranous domain of NHE1, CD44, CD43, and ICAM-2 [16],[20] and that these interactions regulate cell migration and contractility, as well as focal adhesion turnover [21],[22]. The interaction between ion transporters, as integral membrane proteins, and the cytoskeleton mediates the transduction of contractile forces generated from within the cell to the extracellular matrix and promotes migration. However, the mechanistic action of NKCC1 on cell contractility and focal adhesion dynamics in the context of GB cell migration and invasion are entirely unknown.

Activation of NKCC1 transport activity requires phosphorylation of key threonine residues in the NKCC1 N-terminal domain [23]. Phosphorylation of NKCC1 is mediated by at least three members of a novel family of unusual kinases that lack a key lysine in their catalytic domain, the WNK kinases (With No K-lysine) [24],[25]. These kinases have been implicated in the pathogenesis of hypertension and epilepsy [26],[27]. Of these, WNK3 is the most abundantly expressed in the brain [28]. Interestingly, WNK1 is a substrate for Akt-mediated phosphorylation [29]. Hence, it is possible that Akt may regulate NKCC1 activity through the regulation of the WNK kinases. Intracellular signaling pathways, such as phosphoinositide 3-kinase (PI3K)-Akt, are frequently altered in GBs [30]. Akt is able to regulate various cellular functions through phosphorylation of a conserved substrate sequence, and altered regulation of this pathway can lead to aberrant cell behavior, such as increased proliferation and migration [31]–[33]. Importantly, intracellular signaling pathways of promigratory factors such as epidermal growth factor (EGF) [34]–[36], and integrin signaling pathways converge on Akt, modulating cell processes such as cell cycle, apoptosis, and migration [37]. PI3K, the activator of Akt, is thought to be critical in mediating both chemotactic and random cell migration [38]. Therefore, the regulation of NKCC1 by the interaction between Akt signaling and WNK kinases may be important in determining the invasive properties of GB cells.

To further our understanding of the role of NKCC1 in GB cell migration and invasion, we investigated (1) whether the expression of NKCC1 in human tumors correlates with tumor grade, (2) whether NKCC1 affects cell contractility and migration, (3) whether NKCC1 can have an effect on the interaction between the cells and the cells' adhesion substratum, and (4) whether a signaling mechanism involved in the regulation of NKCC1 by promigratory factors exists in GB cells. We found that NKCC1 expression indeed correlates with in vivo glioma aggressiveness and that the transporter activity modulates migration speed and invasiveness of cells derived from various human GBs. Furthermore, we show that NKCC1 expression affects GB cell traction forces, possibly by regulating focal adhesion dynamics. Moreover, the regulation of NKCC and KCC transport by WNK3 may determine the invasive behavior of GB cells. Additionally, we show evidence of NKCC1 phosphorylation regulation by Akt through WNK3 phosphorylation upon stimulation with a promigratory factor, EGF. This suggests an important link between the activation of WNK3 by Akt as well as changes in the activity of ion transport systems in glioma cells. Taken together, these findings strongly suggest that ion transport regulation might be integrated into the control of glioma cell invasiveness in a complex fashion that extends beyond regulation of cell volume and involves the interplay between cell adhesion and growth factor signaling. The understanding of these complex interactions may assist in the design of novel therapeutic strategies.

## Results

### NKCC1 Is Essential for Glioma Cell Invasion

**Figure 1:**
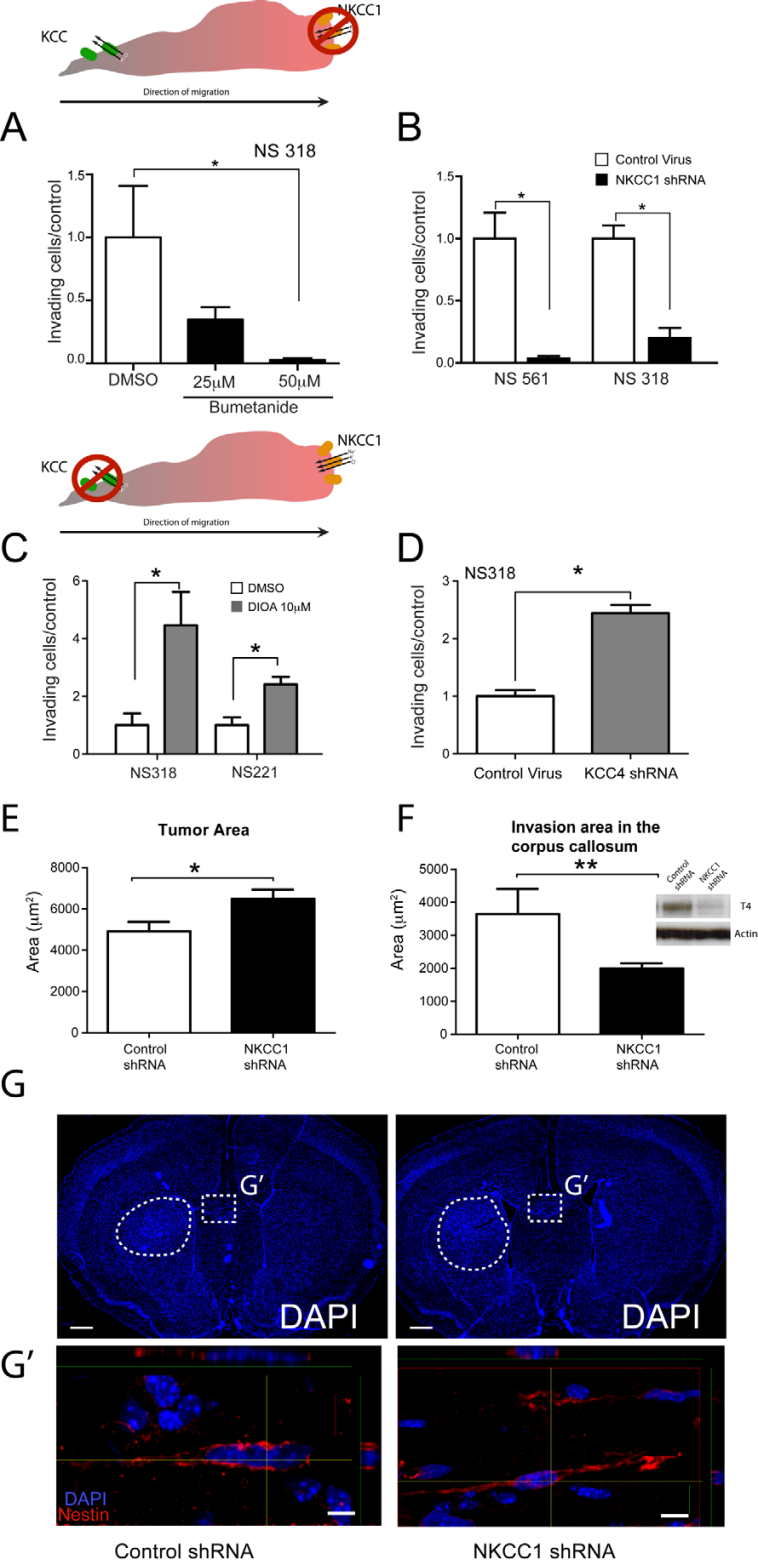
NKCC1 activity is necessary for GB cell invasion in vitro and its inhibition leads to formation of less invasive tumors in vivo. Quantification of transwell invasion assays of primary-cultured GB cells exposed to increasing doses of the NKCC1 inhibitor bumetanide (A) or transduced with NKCC1 shRNA (B); exposed to 10 µM of the KCC inhibitor DIOA (C) or stably transduced with KCC4 shRNA (D). Insets show schematic representation of the experimental design in (A) and (D). (E–F) Orhtotopic in vivo tumors formed by NKCC1shRNA cells were significantly larger and less invasive than control cells. Inset shows NKCC1 knockdown by protein expression. (G) Representative images of DAPI-stained coronal sections of mouse brains, after the implantation of control shRNA (left panel) or NKCC1 shRNA (right panel) cells. (G′) Confocal images of human-specific Nestin positive cells migrating across the corpus callosum at the area in the dotted square in (G). These results suggest that NKCC1 expression is necessary for efficient GB cell migration in vivo. Scale bars, 500 µm in low magnification panels and 20 µm in high confocal images panels. Bars represent mean ± SEM. * *p* value<0.05; ** *p*<0.005.

Prior data implicating NKCC1 in GB invasiveness were based on established, model GB cell lines, rather than primary cells or tissues. We therefore first evaluated and characterized whether primary cells isolated from human GB indeed supported the role of NKCC1 in invasiveness as suggested previously [13]. We assayed invasiveness using the transwell invasion assay in the presence or absence of the NKCC1 inhibitor bumetanide [39]. Inhibition of NKCC1 transport in various primary human glioma cells exposed to 25 and 50 µM of bumetanide led to a dose-dependent decrease in the number of invasive cells (Figure 1A and Figure S1A). Significant inhibition of invasion was seen in GB cells tested at a concentration of 50 µM (Figure S1B), a concentration at which bumetanide does not exhibit considerable non-specific effects on other cation-chloride transporters [40],[41]. To further examine whether the effect of bumetanide on cell invasion is due to inhibition of NKCC1, we performed stable knockdown of NKCC1 using lentiviral particles carrying NKCC1 shRNA. Knockdown of NKCC1 in GB cells (NS561, NS567, NS501, and NS318) was successfully established in 4 GB cell lines and the efficiency of knockdown was assessed by immunoblot of whole cell lysates of these GB cells (Figure S1C). We confirmed that, as previously shown by Haas and colleagues [13], knockdown of NKCC1 significantly reduced the invasiveness of all these cells (Figures 1B, S1D). Taken together, these data suggest that NKCC1 may indeed play a role in invasiveness of primary GB cells, supporting prior results [13] obtained in non-primary cell cultures.

**Figure 2:**
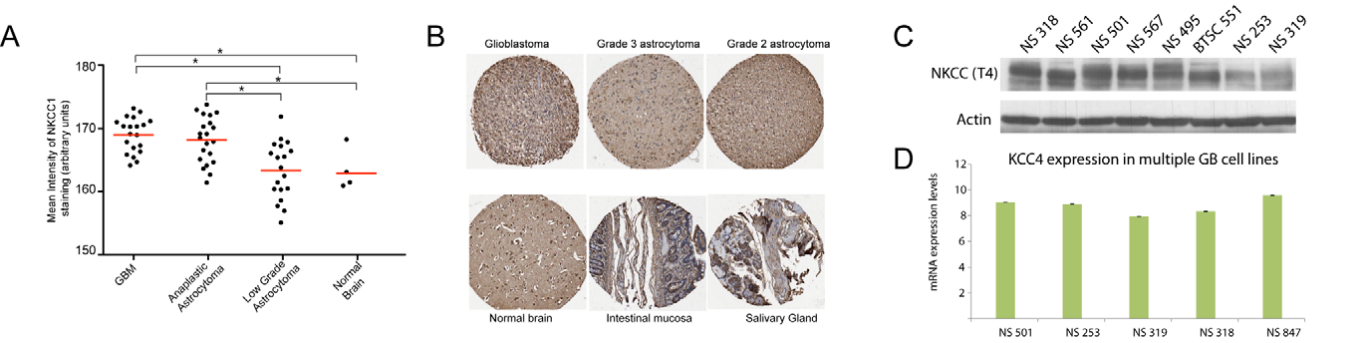
NKCC1 is highly expressed in GB tissue samples and primary human GB cells. (A) Quantification of NKCC1 immunoreactivity in a tissue microarray (TMA) containing samples of multiple glial tumors of different grades. The quantification was done using FRIDA software [100]. Red lines represent mean immunoreactivity levels. (B) Representative images of NKCC1 immunohistochemistry in tissue cores from the TMA including glial tumors of different grades, normal brain, and epithelial tissues, which express NKCC1 in the apical surface of epithelial cells as a positive control. (C) Immunoblot showing NKCC1 expression in multiple glioma cell lines. Information on the number of samples, age, and gender of the patient of origin of each tumor type can be found in Tables S1 and S2. (D) KCC4 expression by real-time PCR in different glioma cell lines. * *p* value<0.001.

Since NKCC and KCC transporters work in a concerted inverse manner to regulate intracellular volume and intracellular chloride concentration ([Cl**^−^**]_i_) [42], we tested whether inhibition of KCC transport, an important Cl^−^ extrusion mechanism, might mimic NKCC1 overexpression and lead to increased invasion. To test this hypothesis we performed transwell invasion experiments in the presence of DIOA (R(+)-Butylindazone), a potent K^+^-Cl**^−^** transport inhibitor that has no effect on NKCC transport activity. Consistent with this hypothesis, inhibition of KCC transport with DIOA resulted in increased cell invasion. This effect was statistically significant in two of the four cell lines tested (NS221 and NS318) (Figure 1C). DIOA is a non-specific inhibitor of KCC co-transporters, which may be the cause of a heterogeneous effect on GB cells observed. To avoid this confounding result we induced the genetic knockdown of KCC4 (Figure S1E), a KCC family member implicated in cervical and ovarian cancer invasiveness [43]. KCC4 had similar expression levels in the cell lines used for the experiments (Figure 2D). Knockdown of KCC4 in NS318 showed a significant increase in the number of invading cells (Figure 1D). These data suggest that KCC transport inhibition could lead to an increase in [Cl**^−^**]_i_ promoting invasive behavior of GB cells.

### NKCC1 Silencing Leads to Decreased GB Stem Cell Invasiveness In Vivo

It is thought that GB tumor stem cells may be the core component of the invasive cell population [44]. Therefore, in addition to invasiveness of primary GB cells in vitro, we explored the role of NKCC1 in the invasion of primary brain tumor stem cells (BTSC) in vivo. Tumor area and area of invasion in the corpus callosum were then quantified to evaluate differences in tumor size and invasive ability of BTSCs carrying the control shRNA as well as BTSCs carrying NKCC1 shRNA. We found that tumors generated after the implantation of BTSCs with control shRNA were significantly smaller than tumors generated with the NKCC1 shRNA harboring BTSC line (Figure 1E). Consistent with previous results by Haas and colleagues using commercial GB cell lines [13], the invaded area in the corpus callosum of mice that were implanted with BTSCs carrying the control shRNA was significantly larger than that of mice implanted with BTSCs carrying NKCC1 shRNA (Figure 1F). NKCC1 knockdown did not affect the proliferative potential of the BTSCs injected in vivo (Figure S2). These results suggest that NKCC1 may be an important determinant of primary and GB stem cell invasiveness, in congruence with prior suggestions based on commercial GB cell lines [13].

### NKCC1 Protein Expression in Human Gliomas Correlates with Tumor Grade

To evaluate the potential clinical importance of NKCC1 in glioma invasion in vivo, we characterized NKCC1 expression in a large array of glioma tissue samples using a tissue microarray (TMA) containing several tumors of different grades ranging from World Health Organization (WHO) Grade II to WHO Grade IV (Table S1). The results revealed that NKCC1 protein expression was significantly higher in GB and anaplastic astrocytoma (AA) tissue samples compared with expression in Grade II astrocytomas and normal brain (Figure 2A and 2B). Epithelial tissues included in the TMA were used as positive controls (intestinal mucosa and tissue from the distal collecting duct in the kidney) (Figure 2B). As a corollary to this analysis and a complement to the results in Figure 1, we characterized the expression levels of NKCC1 protein in multiple primary human GB cells and found that all cell lines tested showed substantial expression of NKCC1 (Figure 2C). The data obtained from this set of samples showed that NKCC1 protein expression indeed correlates with glioma grade, in that tissues from GB and AA expressed higher NKCC1 protein levels than low-grade astrocytomas and normal brain. This correlation between NKCC1 expression with glioma grade suggests that NKCC1 may contribute to the increased invasiveness of high-grade tumors.

### NKCC1 Knockdown Decreases the Speed of GB Cell Migration on Nanopatterned Surfaces

**Figure 3:**
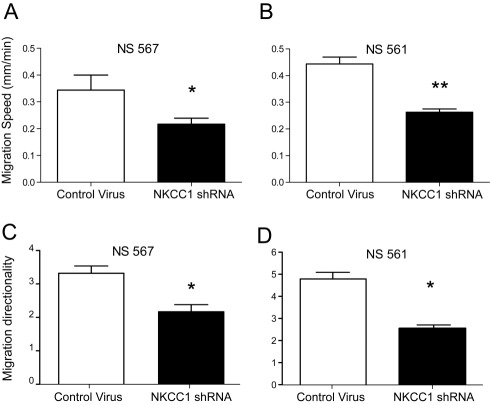
NKCC1 activity is necessary for GB cell migration on a nanopatterned substrate. Quantification of migration speed (A–B) and directionality (C–D) of two different GB cell lines stably transduced with NKCC1 shRNA. NKCC1 shRNA-transduced cells show a decreased migration speed and directionality when compared to control virus-transduced cells. Bars represent mean ± S.E.M. * *p* value<0.05; ** *p* value<0.001. Scale bar represents 50 µm.

Our results so far strongly suggest that NKCC1 may indeed be an important determinant of GB cell invasion. While all prior analyses attempted to link the role of NKCC1 in cell migration to its role as a cell volume regulator [11],[13],[14],[45], we examined whether NKCC1 plays an essential role in the regulation of polarization of cell morphology and migration of GB cells. We were particularly interested in whether NKCC1 might affect cell migration and how this migratory behavior may depend on the mechanical cues mimicking the extracellular matrix components. In this study we employed nanoscale grooves to analyze the migratory behavior of glioma cells. Our substrate mimics ECM features, such as myelinated fiber tracts, upon which brain cancer cells have been shown to migrate [46],[47]. This model offers the advantage of allowing biased cell migration along the nano-ridges of the textured surface that can be quantified in terms of cell speed and migration (Figure S3A–C). We found a significant reduction in the cell migration speed of human primary GB cells stably transduced with NKCC1 shRNA (Figure 3A–B). Similarly, a significant decrease in migration speed was observed when GB cells were treated with bumetanide (Figure S3D). Migration directionality was quantified by measuring the ratio of cell movements parallel to the ridges on the pattern versus those that were perpendicular to the pattern. This metric tended to correlate with the speed of migration, showing significant decreases in directionality for cells expressing NKCC1 shRNA (Figure 3C–D). Overall, GB cells stably transduced with NKCC1 shRNA displayed a lower speed of migration and showed more random migration as demonstrated by the decrease in directionality.

### NKCC1 Deficiency Increases the Size of Focal Adhesions and Decreases Net Contractile Moments in GB Cells

**Figure 4:**
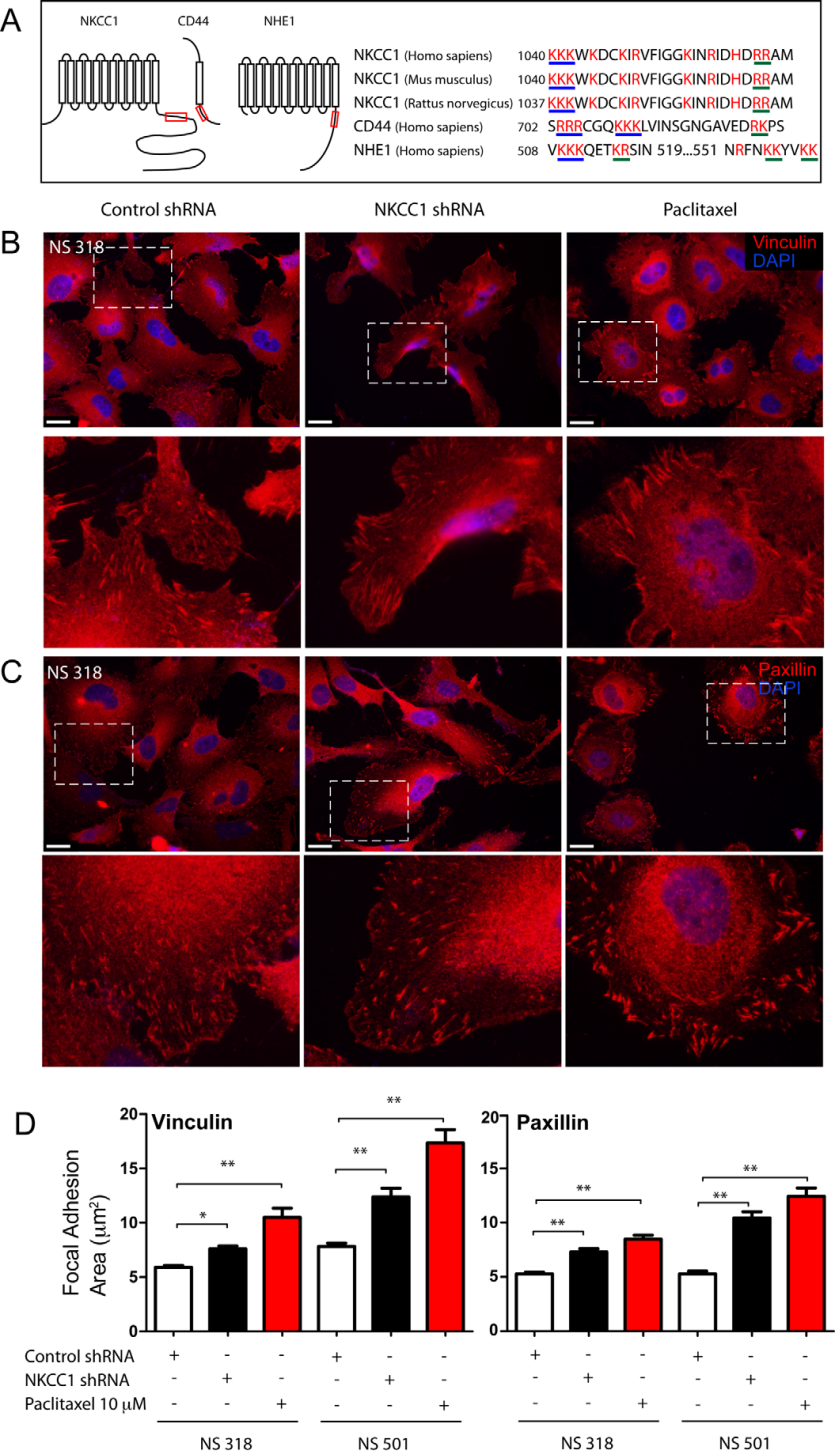
NKCC1 knockdown increases the size of focal adhesions in primary human GB cell lines. Nascent focal adhesions have a smaller area and are primarily responsible for generating traction and participate in generating contractile forces that allow cells to move. Mature focal adhesions have a larger area and are responsible for remodeling the extracellular matrix during migration. Primary human GB cells immunostained for focal adhesion proteins vinculin and paxillin. (A) Left panel, schematic representation of NKCC1, CD44, and NHE1 showing the localization of putative ezrin-radixin-moesin binding domains (red boxes) in the juxtamembranous intracellular domains of these proteins. Right panel, alignment of the protein sequences of NKCC1 of human (NP_001037), mouse (NP_033220), and rat (NP_113986) with the sequence of CD44 (NP_000601) and NHE1 (NP_003038), which have been shown to bind ezrin-radixin-moesin proteins to anchor the actin cytoskeleton to the plasma membrane. In these protein sequences, the positive amino acids such as lysine (K) and arginine (R) are highlighted in red; groups of three positive amino acids are underlined in blue and groups of two positive amino acids are underlined in green. (B–C) NS 318 control shRNA (left panel), NKCC1 shRNA (middle panel), and wild-type treated with 5 µM paclitaxel (right panel) stained with an anti-vinculin antibody (B) and anti-paxillin antibody (C) to visualize focal adhesions (lower panels in B and C show an amplification of areas within the squares). (D) Bar chart of the quantification of focal adhesion area stained with vinculin (*left panel*) and paxillin (*right panel*) antibodies. Quantification of vinculin staining NS318 control shRNA *n* = 23 cells, NS 318 NKCC1 shRNA *n* = 26 cells, NS 318 control shRNA+paclitaxel *n* = 22 cells, NS 501 control shRNA *n* = 20 cells, NS 501 NKCC1 shRNA *n* = 20 cells, and NS 501 control shRNA+paclitaxel *n* = 10 cells. Quantification of paxillin staining NS 318 control shRNA *n* = 21 cells, NS 318 NKCC1 shRNA *n* = 15 cells, NS 318 control shRNA+paclitaxel *n* = 21 cells, NS 501 control shRNA *n* = 12 cells, NS 501 NKCC1 shRNA *n* = 24 cells, and NS 501 control shRNA+paclitaxel *n* = 10 cells. * *p* value<0.05; ** *p* value<0.01. Scale bars represent 50 µm.

The cell migration data indicated that NKCC1 can directly or indirectly affect cell motility, but the mechanism of how an ion transporter can be involved in this process is not immediately apparent. It is therefore of interest to note that at least some ion transporters have been reported to associate with the ERM complex to anchor actin to the plasma membrane, affecting cell migration [16],[19]. The ERM complex proteins bind to clusters of positive amino acids such as lysine (K) and arginine (R) in proteins that are known to bind ERM proteins and to serve as anchors for the actin cytoskeleton such as CD44, CD43, and ICAM-2 [20]. Also, NHE1, a Na^+^-H^+^ exchanger, acts as an anchor for the cytoskeleton in migrating cells, through the interaction with ERM proteins [16]. Based on these data, we studied the sequence of the juxtamembrane carboxy-terminus domain of human NKCC1 and found clusters of positively charged amino acids identical to those found in other ERM binding proteins. These clusters of positive amino acids are conserved in the human, mouse, and rat NKCC1 sequences (Figure 4A). These amino acids may be important in the interaction between ERM proteins and NKCC1 and may be similar to other ERM-integral membrane protein binding [20].

To assess the possibility that NKCC1 may affect GB cell migration through a mechanism other than cell volume regulation, we compared the size of focal adhesions formed by NKCC1 knockdown cells and cells transduced with the control shRNA. Focal adhesions were stained with an antibody against vinculin and paxillin, cytoskeletal proteins that are part of focal adhesions that also regulate mechanical coupling of the cytoskeleton to the extracellular matrix (ECM). We observed small, thin, and elongated focal adhesions primarily in the extending processes in control virus shRNA cells, whereas in NKCC1 shRNA cells, focal adhesions were much larger (Figure 4B–C and Figure S4), indicative of focal adhesion maturation [22],[48]. The area of focal adhesions was significantly larger in NKCC1 shRNA cells when compared to control virus cells (Figure 4D). The increased focal adhesion area was also seen when we used paclitaxel (a drug that stabilizes microtubules dynamics and disrupts focal adhesion formation) as a positive control for this experiment [49]. These results suggest that NKCC1 expression not only regulates cell volume but may also be important in modulating focal adhesion dynamics and maturation.

**Figure 5:**
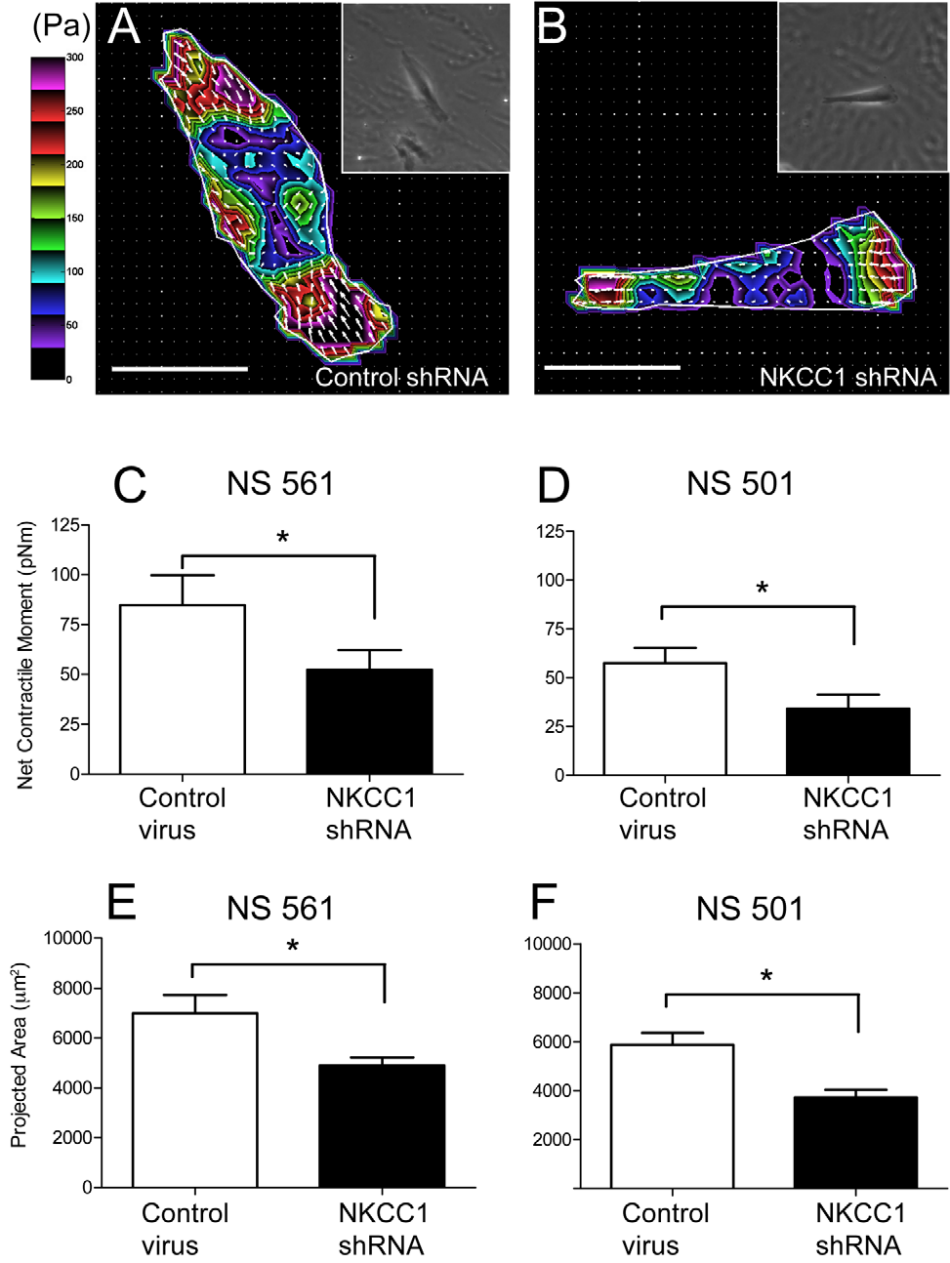
NKCC1 knockdown decreases the net contractile moment and projected area in primary human GB cell lines. (A and B) Representative traction maps of GB cells stably expressing control shRNA or NKCC1 shRNA, respectively. The white line shows the cell boundary. Colors show the magnitude of the tractions in Pascal (Pa). Arrows show the direction and relative magnitude of the tractions. Scale bars represent 50 µm. Inset, phase contrast images of the respective cells on the elastic gel. Computed net contractile moment of GB cells expressing control shRNA or NKCC1 shRNA in (C), NS 561 (control shRNA *n* = 15 cells, NKCC1 shRNA *n* = 14 cells, *p* = 0.024) and (D) NS 501 (control shRNA *n* = 13 cells, NKCC1 shRNA *n* = 12 cells, *p* = 0.005). Net contractile moment is expressed in pico-Newton meter (pNm). Measurement of the projected cell area in µm^2^ of (E) NS 561 (control shRNA versus NKCC1 shRNA, *p* = 0.01) and (F) NS 501 (control shRNA versus NKCC1 shRNA, *p* = 0.001). Data are presented as geometric mean ± SEM in log transformation.

Cells exert traction forces on their environment during migration and invasion in response to different mechanical and chemical cues in the extracellular matrix. These forces are applied through points of cell adhesion via focal adhesion-mediated integrin-ECM connections [21],[22]. To evaluate whether the increase in size of focal adhesions after NKCC1 depletion had a functional effect on the generation of contractile forces by GB cells, we quantified cell traction forces exerted by adherent living GB cells (control virus versus NKCC1 shRNA). We found that NKCC1-deficient cells exerted significantly lower cell traction forces than control virus cells (Figure 5A–B). Compared to control virus cells, NKCC1 shRNA cells exhibited approximately a 40% decrease (NS501, 44% decrease; NS561, 37% decrease; *p*<0.002, nested ANOVA) in net contractile moments, which is a scalar measure of cell contractile strength (Figure 5C–D, Figure S5). No within-group differences existed between both tested cell lines.

**Figure 6:**
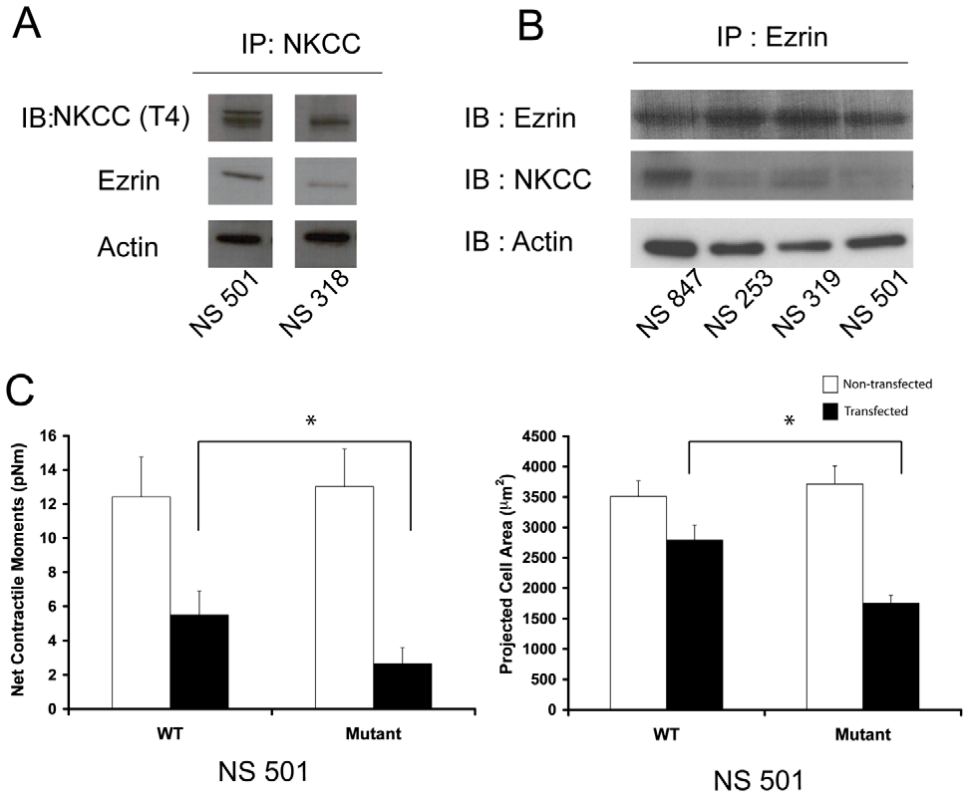
NKCC1-Ezrin association affects net contractile moments and projected cell area. (A) Immunoprecipitation of NKCC1 in two GB cell lines shows that Ezrin and actin are associated with NKCC1. (B) Immunoprecipitation of Ezrin pulls down NKCC1 in several GB cell lines. (C) Cells that overexpress Ezrin-binding null NKCC1 generate lower contractile moments and have lower surface area. Black bars represent the transfected cells, and white bars represent the untransfected cells. Measured cells in the WT group (*n* = 16 untransfected cells; *n* = 11 transfected cells), and measured cells in the mutant group (*n* = 17 untransfected cells; *n* = 12 transfected cells). Bars represent mean ± S.E.M. * *p* value<0.001.

To further support the interaction of NKCC1 and ERM proteins, we immunoprecipated endogenously expressed NKCC1 and probed the immunoprecipated lysate with an antibody against Ezrin. We found that endogenous Ezrin associates with immunoprecipitated NKCC1. These results strongly suggest that in primary human GB cells, Ezrin is an NKCC1 binding partner. As expected, actin, a binding partner of Ezrin, also co-immunoprecipitated with NKCC1 (Figure 6A). We also performed the reverse experiment where Ezrin was immunoprecipitated and then probed for NKCC1 on the immunblot. In multiple primary human GB cell lines, we found that after performing immunoprecipitation of Ezrin, NKCC1 was also pulled down (Figure 6B). As expected, actin was also co-immunoprecipitated.

To assess whether the association of NKCC1 and Ezrin is important for the generation of cell traction forces, we mutated the two clusters of basic amino acids found in the juxtamembranous domain of NKCC1 (putative Ezrin-binding sites) and measured the functional consequences in GB cells. We found that the net contractile movements of cells expressing the Ezrin-binding null NKCC1 were significantly lower than cells expressing wild type-NKCC1 (Figure 6C). Furthermore, cells expressing the Ezrin-binding null-NKCC1 had a lower projected cell area than cells expressing wild-type NKCC1 (Figure 6D).

Thus, the absence of NKCC1 expression may lead to the formation of more mature focal adhesions. In turn, these mature focal adhersions may further enhance the adhesion of cells to the substratum, which decreaces the migration speed of NKCC1 shRNA cells, as observed above. Mature focal adhesions do not participate in the generation of contractile forces in migrating cells; rather, they participate in anchoring cells to the substrate [21]. On the other hand, nascent adhesions apply forces to the substratum to drive cell movement [21]. Hence, our results suggest that NKCC1 affects cell-ECM interactions by stimulating higher traction force generation and lower substratum adhesion, thus enhancing cell motility in a synergistic fashion.

### NKCC1 Is Expressed at the Distal Edge of Extending Processes of Primary Human GB Cells

**Figure 7:**
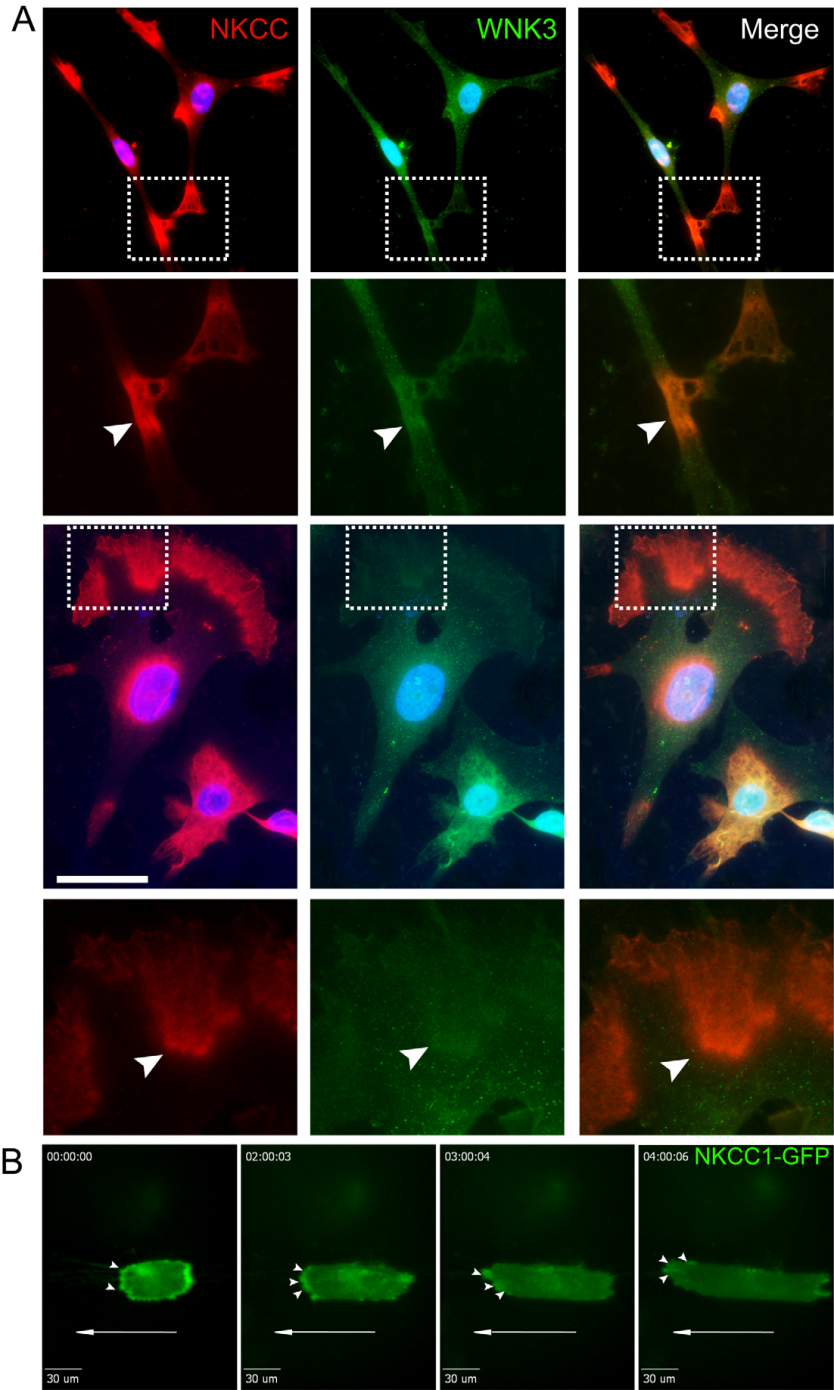
NKCC1 localizes to the extending processes of migrating cells with directional polarization. (A) Images of NS 253 cells immunostained with T4 antibody (red, left panel), WNK3 antibody (green, center panel), and DAPI (blue). The right panel shows co-localization of NKCC1 and WNK3 immunoreactivity. Below, high magnification images of the area in the dotted boxes are presented for more detail. Areas of colocalization are pointed out using arrowheads. Scale bars represent 50 µm. (B) Images of NS 561 cells expressing NKCC1-GFP protein migrating on a nanopatterned substrate shows localization to the advancing edge of extending processes in migrating cells at time-point 0 min (left panel), 2 h (center left panel), 3 h (center right panel), and 4 h (right panel).

The pronounced effect of NKCC1 expression on focal adhesion formation and cell migration suggests the importance of its partial intracellular localization. We initially approached this issue by performing immunocytochemistry experiments on multiple GB cells. We found that all GB cells had a polarized subcellular expression of NKCC1. In cells that appeared to have a more stationary phenotype with multiple projections, the expression of NKCC1 was primarily correlated with these projections (Figure 7A and Figure S6). Specifically, NKCC1 was localized either to the apparent leading edge of a moving cell or to its rear, frequently in a mutually exclusive pattern (Figure 7B and Figure S6). Expression of NKCC1-EGFP fusion protein in GB cells supported that NKCC1-EGFP expression was mainly localized to the plasma membrane of the extending processes confirming the results obtained by immunofluorescence (Figure 7B, Figure S7, and Video S1 and Video S2). In cells spreading on nano-structured substrata, NKCC1-EGFP localization oscillated between the two transiently existing edges, before a prominent single edge was formed. Furthermore, when using immunocytochemistry, we examined the sub-cellular localization of WNK3, a serine/threonine kinase that regulates the transport activity of NKCC1 through phosphorylation [26],[27],[50]. We observed partial co-localization of WNK3 immunoreactivity with NKCC1 immunoreactivity in the edges of extending processes (Figure 7A and Figure S6). These findings suggest that the cellular localization of NKCC1 is spatially heterogeneous during GB cell migration. Although the localization patterns were diverse in different cell states, the overall pattern that emerged from this analysis was that NKCC1 is associated with extending processes of the cell. This finding correlates with the suggestion that NKCC1 is important in the formation of new focal adhesions and controlling existing focal adhesions and active cytoskeletal components.

### NKCC1 Is Activated by EGF Through Akt-Mediated WNK3 Phosphorylation

**Figure 8:**
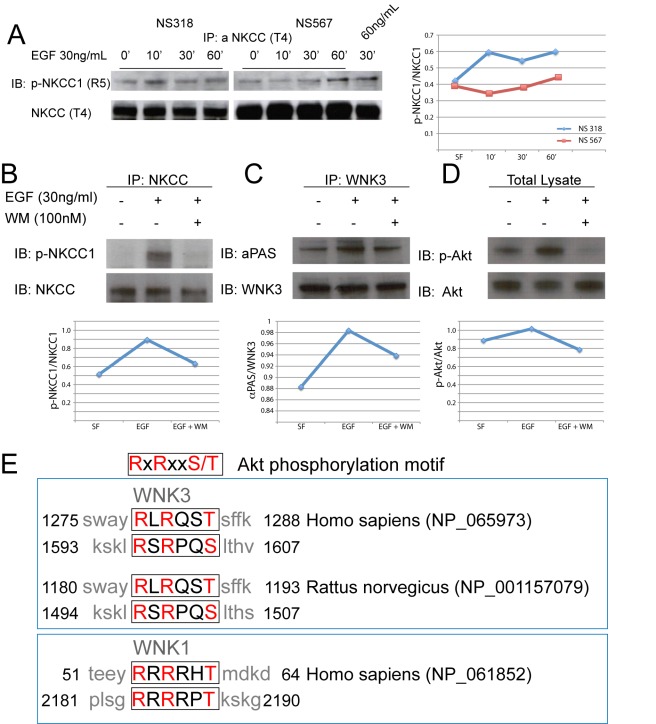
EGF promotes phosphorylation of NKCC1, and activation of the PI3K-Akt pathway is necessary for EGF-mediated WNK3 phosphorylation. (A) Treatment of GB cell lines NS 318 and NS 567 with EGF (30 ng/ml) stimulates phosphorylation of NKCC1. Exposure of cells to EGF (30 ng/ml) for 10, 30, and 60 min show a time-dependent course of NKCC1 phosphorylation. Also, exposure of NS 567 to 60 ng/ml of EGF shows higher levels of phosphorylation than phosphorylation levels at the same time point at 30 ng/ml showing a dose-dependent effect. A line plot is presented with the quantification of the ratio of p-NKCC1/NKCC1. (B) Activation of PI3K is necessary for phosphorylation of NKCC1 after stimulation of HEK-293 cells with EGF. HEK-293 cells were serum starved overnight and were incubated with wortmannin (WM) for 30 min prior to stimulation with EGF for 30 min. Total cell lysate (150 µg) was immunoprecipitated with T4 antibody before immunoblotting with anti-phospho NKCC1 antibody (top panel) or T4 antibody (bottom panel). (C) Activation of PI3K is necessary for increased phosphorylation of WNK3 after stimulation with 30 ng/ml EGF. After overnight serum starvation, HEK-293 cells were incubated with WM for 30 min prior to stimulation with EGF for 30 min. Total cell lysate (150 µg) was immunoprecipitated with WNK3 antibody before immunoblotting with anti-phosphorylated Akt substrate (αPAS) antibody (top panel) or WNK3 (bottom panels). (D) Total cell lysate samples (25 µg) were also resolved by SDS-PAGE and blotted with phospho-Akt (threonine 473, top panel) and Akt (bottom panel) antibodies to show inhibition of Akt phosphorylation after PI3K inhibition. A line plot is presented with the quantification of the ratio of p-NKCC1/NKCC1, αPAS/WNK3, and p-Akt/Akt. (E) Akt phosphorylation motif, top panel. Middle panel shows the alignment of the sequences of rat and human WNK3 showing conservation of the Akt phosphorylation motif. Bottom panel shows sequence of WNK1, which is phosphorylated by Akt (bottom panel) [29]. Conserved residues are highlighted in red letters.

The aforementioned data suggest that NKCC1 transport activity is important for glioma cell migration and invasion, at least in part through direct regulation of the cytoskeletal and ECM-cell adhesion dependent processes. NKCC1 transport activity is known to be regulated through phosphorylation and de-phosphorylation events mediated by members of the novel serine/threonine kinase family WNKs [26]. NKCC transport is activated by stimulation with EGF in corneal epithelial cells [51]. It is well established that EGF promotes astrocytic [34] and glioma cell migration [36],[52],[53]. Thus, we examined the effect of EGF on the phosphorylation of NKCC1 as an indication of NKCC1-activation using an NKCC1 phospho-specific antibody [23]. After stimulating glioma cells with EGF, NKCC1 phosphorylation increased in a time-dependent and dose-dependent manner in NS318 and NS567 cells (Figure 8A). To gain insight into the regulation of phosphorylation of NKCC1 in an unbiased cellular system, we stimulated HEK-293 cells with EGF in the presence or absence of wortmannin (WM), a PI3K inhibitor. After exposure of HEK-293 cells to EGF, NKCC1 phosphorylation increased significantly. However, in the presence of WM, EGF-induced NKCC1 phosphorylation was blocked (Figure 8B). These findings together demonstrate that the EGF-induced increase in phosphorylation of NKCC1 requires activation of the PI3K-Akt pathway.

The activity of cation-chloride cotransporters is regulated in a coordinated manner by the novel family of serine-threonine kinases WNK [50]. WNK3 promotes phosphorylation and activation of NKCC1 transporters while promoting phosphorylation and inactivation of KCC transporters [50]. It has also been shown that WNK1, another member of the WNK family, is phosphorylated and activated by Akt (protein kinase B) [29],[54]. Therefore, we decided to test if Akt phosphorylates WNK3 after stimulation with EGF. We immunoprecipitated total WNK3 from HEK-293 cells exposed to serum-free media, and we stimulated with EGF, or EGF in the presence of the PI3K inhibitor WM. Samples were immunoblotted with an antibody that recognizes phosphorylated Akt substrates (αPAS antibody) (Figure 8C). Data obtained from this experiment showed a basal phosphorylation level of WNK3, which increases with exposure to EGF. This increase in phosphorylation is repressed by inhibition of PI3K with WM, suggesting that Akt phosphorylates WNK3. The same samples were immunoblotted against phosphorylated Akt, and as expected, we found that WM also inhibited the phosphorylation of Akt (Figure 8D). In silico analysis of the protein sequence of WNK3 revealed that two putative Akt phosphorylation motifs are present and are conserved in available WNK3 protein sequences of the human and rat, as previously found in WNK1 (Figure 8E) [54]. These findings indicate that NKCC1 may be activated by factors that stimulate migration of astrocytic or glioma cells, such as EGF via kinases of the WNK family, a family of kinases that have been shown to regulate the transport activity of multiple members of the SLC12A family of transporters.

## Discussion

The nearly universal recurrence of GB after surgical resection is largely due to invasion of glioma cells into healthy brain tissue and presents a major impediment in the improvement of GB patient survival. In this study, we tested the hypothesis that NKCC1 expression and transport activity are crucial elements of cell migration and invasion in primary human GB cell lines. Here we provide evidence for the participation of NKCC1 in the migration and invasion of primary human glioma cells, highlighting its possible role in anchoring the actin cytoskeleton to the plasma membrane. Additionally, through this anchoring, NKCC1 mediates transduction of the cellular contractile forces to focal adhesions that interact with the extracellular matrix. These in vitro findings were confirmed functionally in an in vivo model using primary human BTSCs, where their invasion was decreased significantly after NKCC1 knockdown. Given these findings, NKCC1 inhibition could potentially be used in the clinic to improve glioblastoma treatment, given that Bumetanide (a commonly used diuretic, FDA-approved) decreases the invasive potential of glioma cells in vivo [13]. This could potentially improve surgical resection of the tumor mass, as tumor cells lacking NKCC1 activity would form less invasive tumors.

Our work shows that NKCC1 protein expression in multiple glioma samples is higher in high-grade gliomas such as GB and anaplastic astrocytomas. Inhibition of NKCC1 transport pharmacologically, as well as genetic inhibition of NKCC1 expression, decreases invasion of multiple primary human GB cell lines. These results are in accordance with previous findings using commercial human glioma cell lines [13]. Our data further indicate that migration speed of GB cell lines in a 2-D nanopatterned substrate is decreased by pharmacological inhibition and by shRNA-based silencing of NKCC1 expression. Interestingly, pharmacological and genetic inhibition of the K^+^-Cl^−^ cotransporters leads to a more invasive behavior of GB cells in vitro. Moreover, our results suggest that NKCC1 may affect the morphology of focal adhesions, perhaps due to a putative ERM binding motif in the cytoplasmic domain of NKCC1. We also found that NKCC1 is located at the extending processes of GB cells and that NKCC1 polarization may precede migration towards the direction of this pole. Furthermore, exposure of GB cells to EGF, a factor that promotes migration and invasion of normal and tumor cells [34]–[36],[55],[56], induces phosphorylation (activation) of NKCC1 through PI3K-Akt-WNK3 pathway.

The concerted action of local anchoring of the actin microfilaments to the plasma membrane and volume regulation may be important for the polarization of cells during migration. Coupling the actin cytoskeleton to the plasma membrane is essential for the regulation of cell morphology and migration [6]. Our immunocytochemistry and live cell imaging experiments using a GFP-NKCC1 fusion protein demonstrate that NKCC1 is localized to the extending processes of migrating GB cells. During the migratory process, cells acquire a polarized morphology where actin, integrin receptors, and ion transporters among other proteins, become asymmetrically distributed in the cell. Some examples of ion transporters that show a polarized localization to the leading edge of the cell include NHE1 and AE2 [9],[16],[17], which also have K^+^ channels that are polarized to the rear end of the cell [57]. Ezrin-radixin-moesin (ERM) proteins bind actin filaments and anchor them to integral plasma membrane proteins. Some of these integral membrane proteins include NHE1, CD44, and intercellular cell adhesion molecule 2, which have ERM binding motifs (ICAM-2) [16],[18]. The ERM binding motif consists of clusters of positive amino acids, such as lysine and arginine residues in juxtamembranous intracytoplasmic domains of these proteins. By analyzing the peptide sequence of NKCC1, we found clusters of lysine and arginine residues in the N-terminal cytoplasmic domain that are conserved across mammalian species, which may bind ERM proteins. Indeed, we found that NKCC1 is able to bind to Ezrin and actin with our co-immunoprecipitation assay. The generation of advancing membrane protrusions is necessary for migrating cells to achieve cell translocation. ERM protein binding to NHE1 is necessary during migration to promote extension of advancing processes to anchor the cytoskeleton [16],[17]. Our results show that NKCC1 is polarized to the extending processes of migrating glioma cells; therefore, it is likely that NKCC1 is necessary during migration to anchor the cytoskeleton, aiding in the extension of lamellipodia, and mediate local volume changes at the same time [14].

NKCC1 expression may be an important determinant of the response of migrating cells to external physical cues. The extracellular matrix surrounding cells presents topographical features ranging from nanometers (nm) to microns (µm) and affects cell behavior. For example, collagen fibrils form with diameters from 20–200 nm and influence cell polarity and migration through “contact guidance” [58]–[65]. Recent studies have employed more intricate substrates presenting nanoscale features (e.g., grooves, ridges, bumps, and pillars) to more closely model the cellular microenvironment [66]–[68]. In this study, we have employed nanoscale features mimicking the ECM found in the brain. These features include myelinated fiber tracts, upon which brain cancer cells have been shown to migrate [47]. It is known that cell migration is governed by many molecular processes, including attachment to the cell substrate. Our nanopattern provides a quasi-3-D platform that can examine these interactions with ECM by examining speed, direction, and morphologies of migrating cells. While cells move in 2-D, they respond to topographical cues from the substrate presented in 3-D [46],[69].

By examining cell migration after genetic changes to NKCC1 expression, we sought changes that might inhibit the overall motility of glioma cells. Our observed changes in migratory behavior upon simulated ECM suggests that these cells will be less migratory and invasive, eventually leading to improved medical outcomes. These changes in migratory behavior were further supported by our experiments employing more classical techniques (e.g., transwell).

It has been shown that formation of actin stress fibers precedes the formation of nascent focal adhesions in the lamellipodium of fibroblasts [70]. However, stress fiber formation depends on the anchorage of actin bundles to the plasma membrane, which has been shown in the interaction of NHE1 with ERM proteins [16]. Nascent adhesions are present at the front of the cell and exert traction forces that lead to cell repositioning [21]. As focal adhesions increase in size, they mature and the traction forces that they exert decrease considerably [21]. In migration studies of cells that do not express focal adhesion kinase (FAK), a major regulator of focal adhesion turnover, it was shown that these cells possess larger focal adhesions and display lower cell spreading [71],[72]. In fact, these observations were also seen in GB cells when NKCC1 is knocked down; NKCC1 knockdown cells display larger focal adhesions and smaller projected cell area than control shRNA cells. These changes in focal adhesion size were accompanied by a decrease in the generation of contractile forces by GB cells. These findings suggest that the localized distribution of NKCC1 to the extending processes plays a role in the modulation of focal adhesion turnover and generation of nascent focal adhesion to maintain cell contractility and traction for efficient migration.

Cell volume changes are expected in migrating cells since alterations in shape during extension and retraction occur throughout migration. Multiple ion transport mechanisms are responsible for regulating and maintaining cellular volume in response to changes in extracellular osmolarity and during cell migration [5]; in addition, ion gradients and local volume changes have been described in migrating cells [73],[74]. These mechanisms include ion transporters such as NKCC1, NHE1, KCC transporters, and also ion channels. For instance, neutrophils undergo an increase in intracellular volume in response to the chemotactic factor N-formylmethionyl-leucyl-phenylalanine; this increase in cell volume and increased migration is blunted by NHE1 transport inhibitors and by exposure to hyperosmolar solutions, suggesting that NHE1-mediated volume increase is necessary for neutrophil migration [7],[75]. Na^+^-K^+^-Cl^−^ transport inhibition has also shown to decrease Madin-Darby canine kidney cell migration [74]. Our results are in accordance with the findings discussed above where inhibition of NKCC transport decreases migration. We also show that NKCC1 knockdown decreases GB cell migration, confirming the effects of pharmacologic inhibitors. Pharmacological inhibitors and shRNA-based approaches may have off-target effects, but the fact that the effect of both on GB cell migration is the same confirms that the results seen by manipulating NKCC1 expression/transport are consistent.

EGFR activation promotes migration of normal neuroblasts, astrocytes, and glioma cells [34],[53],[76]–[78]. EGF signaling affects migration through diverse mechanisms, such as actin polimerization [79],[80], focal adhesion kinase regulation [81]–[83], and matrix metalloproteinase expression [84]. EGF mediates its effects on cell migration and proliferation through activation of its receptor-tyrosine kinase and the various downstream signaling pathways, which include the PI3K-Akt pathway. NKCC1 phosphorylation by WNK3 after activation of the PI3K-Akt pathway supports the hypothesis that NKCC1 activity is necessary for GB cell migration. Furthermore, WNK3 activation after EGF stimulation suggests that phosphorylation and activation of NKCC1 and phosphorylation and inhibition of KCC transporters may result in GB cell migration. Therefore it seems this balance between these opposing transport activities is important in the determination of GB cell invasion.

The PI3K-Akt signaling pathway, among many other cell functions, is central in the control of cell motility and polarization. PI3K is activated by receptor tyrosine kinases and Ras [85]. It modulates these functions by bringing diverse proteins that are able to bind phosphatidyl-inositol triphosphate (PIP3) close to the membrane. A notable example of the proteins that are recruited to the membrane is Akt, which is activated after binding to PIP3 and phosphorylated by 3′-phosphoinositide-dependent kinase 1 (PDK1); AKT is recruited in a polarized manner to the leading edge of the migrating cell membrane [86]. It is well known that PI3K activation mediates cytoskeletal rearrangements and cell polarization through the action of the guanine nucleotide exchange factors (GEFs) [87],[88]. Moreover, PI3K modulates actin polymerization and membrane insertion at the leading edge of a cell by regulating the activity of Arf [89]. Its activation also promotes cell polarization through Rac regulation [90]. It is still necessary to assess if the cytoskeletal rearrangements and cell polarization mediated by PI3K are important in the generation of the partial distribution of NKCC1 to the extending processes of GB cells.

WNK3 regulates ion transport through phosphorylation: it phosphorylates and activates NKCC1 and phosphorylates and inhibits transport of the KCC transporters in a reciprocal manner [27],[50]. Furthermore, Haas et al. have shown that following a hyperosmotically induced decrease in cell volume, WNK3 may regulate NKCC1. Also, the reduced expression of WNK3 by shRNA diminished the ability of glioma cells to migrate in vitro [91]. Our results show that inhibition of NKCC and KCC transport result in opposite effects in GB cell migration. Similar to WNK1, our immunoprecipitation experiments show that EGF induces phosphorylation of WNK3 through Akt [29],[54]. When phosphorylated, WNK3 then phosphorylates NKCC1 and possibly KCC transporters. This mechanism is similar to the mechanism proposed for increased excitability of neurons, where WNK3 signaling is impaired, resulting in GABA-mediated excitation of neurons and seizure activity [27]. Therefore, it is conceivable that WNK3 activation results in activation of NKCC1 and inhibition of KCC transport, causing increased migration of GB cells.

In this study, we show that NKCC1 transport expression and activity are necessary for GB cells to migrate and invade. The mechanism affecting cell contractility that we report in this article may be independent from regulation of volume changes and may be due to regulation of focal adhesion formation and turnover. We also show that EGF regulates NKCC1 phosphorylation through an Akt-WNK3 pathway, linking the PI3K-Akt pathway to the WNK3 kinase. This suggests that WNK3 may have a role in determining GB cell migratory properties. Furthermore, given that NKCC1 is ubiquitously expressed, it is possible that it plays a very similar role in physiological migration such as inflammatory cell diapedesis or neural precursor migration during development, as well as in the process of metastasis of other highly aggressive cancers.

## Materials and Methods

### Cell Lines

Patient samples of glioma tissues were obtained at the Johns Hopkins Hospital under the approval of the Institutional Review Board (IRB). All human brain tumor cell lines were derived from intraoperative tissue samples from patients treated surgically for newly diagnosed glioblastoma multiforme without prior treatment as listed in Table S2. Differentiation potential of cell lines for an in vivo experiment was evaluated by immunohistochemistry against GFAP, TuJ1, and NG2 (Figure S9). Detailed culture methodology has been previously described [92],[93].

### Generation of NKCC1-shRNA Stable Expressing Cell Lines

VSV-G pseudotyped virus was produced by co-transfecting 293T cells with a shRNA transducing vector and two packaging vectors: psPAX2 and pMD2.G. The shRNA sequence used was 5′-TAG TGC TCT CTA CAT GGC ATG GTT AGA AGC TCT ATC TAA GGA CCT ACC ACC AAT CCT C-3′. Seventy-two hours after transduction, cells were cultured in the presence of puromycin for selection of cells expressing the shRNA. Knockdown was assessed by quantitative PCR (Figure S8) and immunoblot (inset in Figure 1F and Figure S1C).

### Cloning of Full-Length Human NKCC1 cDNA, Site-Directed Mutagenesis, Subcloning Into Lentiviral Vector, and Generation of EGFP Fusion Protein

The sequence of human NKCC1 (SLC12A2, accession number NM001046) was amplified by PCR using gene-specific primers. The sequence of the primers employed is as follows: sense, 5′- GCG TGC TGC CGG AGA CGT CC-3′; antisense, 5′- AGT CAC CAT TCG CCA TTG TGA TGT T-3′. The resulting PCR product was cloned into pCR-XL-TOPO (Invitrogen). The cloned sequence was verified in its entirety to confirm the absence of mutations. The EGFP fusion protein was made by cloning the NKCC1 open reading frame into pcDNA3-EGFP using standard cloning procedures. All other procedures are listed in Supplemental Experimental Procedures (Text S1).

### Quantitative Real-Time Polymerase Chain Reaction

Total RNA was extracted from primary glioma cell lines using the RNAeasy kit (Qiagen) and reverse transcribed using the SuperScript III First-Strand Synthesis System for RT-PCR (Invitrogen). The target cDNAs were analyzed using SYBR Green PCR master mix (Applied Biosystems) in a 7300 Real-Time PCR system (Applied Biosystems). For relative quantification, the results obtained were compared to the levels of target mRNA expression present in the control cell line and normalized for GAPDH expression. Primers are listed in Supplemental Experimental Procedures (Text S1).

### Immunoblotting

NKCC, WNK3, Akt, Ezrin, and actin were detected using rabbit and mouse primary antibodies. Detection was done with the appropriate horseradish-peroxidase conjugated secondary antibodies and using the enhanced chemiluminescence reagent (GE Healthcare Life Sciences). Antibodies are listed in Supplemental Experimental Procedures (Text S1).

### Immunoprecipitation

Cell lysates (150 µg of protein) were incubated with anti-NKCC antibody (T4 antibody, 1 µg; DSHB) and anti-Ezrin (cell signaling cat: 3145, 1∶100) overnight at 4 °C on a shaking platform. Indirect immunoprecipitation was done with protein G magnetic beads (Millipore). Proteins were then eluted and denatured in LDS protein loading buffer (Invitrogen).

### In Vitro Invasion Assay

Fifty thousand cells were plated in the top chamber of a matrigel-coated membrane (24-well insert; pore size, 8 mm; BD Biosciences). Cells were plated in medium containing 0.5% of serum, whereas medium with 2% serum was used as a chemo-attractant in the lower chamber. After 48 h cells that invaded were stained and counted for comparison.

### Nanogrooved Pattern Cell Migration Assay

Migration of glioma cells was quantified using a novel directional migration assay using nano-ridges/grooves constructed of transparent poly(urethane acrylate) (PUA), and fabricated using UV-assisted capillary lithography (see Figure S3A–C) [94]. Nanopattern surfaces were coated with laminin (3 µg/cm2). Cell migration was quantified using timelapse microscopy (Video S3). Long-term observation was done on a motorized inverted microscope (Olympus IX81) equipped with a Cascade 512B II CCD camera and temperature and gas controlling environmental chamber. Phase-contrast and epi-fluorescent cell images were automatically recorded under 10× objective (NA = 0.30) using the Slidebook 4.1 (Intelligent Imaging Innovations, Denver, CO) for 15 h at 10–20-min intervals.

### Quantitative Analysis of Cell Migration

A custom-made MATLAB script was used to identify cell boundaries from phase-contrasted images and to measure cell centroid positions. Average individual cell speed was calculated from individual cell trajectories and durations of the image acquisition. Mean squared displacements at various time intervals were calculated using a previously published method [95].

The spindle shape factor was defined as the ratio of the length of maximum cell width (maximal axis) to the minimum value of the cell width in the direction perpendicular to maximum axis, regardless of the orientation with respect to nanogrooves. For each condition, over 60 cells were quantified in total.

For quantitative analysis of cell orientation, cells were fixed and stained for F-actin with phalloidin. The orientation angle of polarized cell was determined by measuring the acute angle between the major axis of the cell and the direction of grooves. More than 100 cells for each group were used to construct the polarization angle distributions with range −90° and 90°. A summary of all the migration assays used is presented in Table S3.

### Fourier Transform Traction Microscopy (FTTM)

The contractile stress arising at the interface between an adherent cell and its substratum was measured with traction microscopy [96]. For each cell analyzed, the traction field was computed using Fourier transform traction cytometry as described previously. The computed traction field was used to obtain the net contractile moment, which is a scalar measure of the cell's contractile strength (Figure S5) [97].

### Immunofluorescence

Cells were fixed in 4% paraformaldehyde in phosphate-buffered saline (pH 7.4) for 1 h and blocked with 10% normal donkey serum in PBS for 1 h. Subsequently, fixed cells were incubated with primary antibody at 4 °C overnight. The preparation was then incubated with Alexa Fluor-conjugated secondary antibodies (Invitrogen) and mounted using Aquamount (VWR). All antibodies and their dilutions are listed in Supplemental Experimental Procedures (Text S1).

### Intracranial GB Cell Injections, Sectioning, and Histochemistry

All animal protocols were approved by the Johns Hopkins Animal Care and Use Committee. In vivo invasion and tumorigenesis of cells expressing NKCC1 shRNA were assessed in 4- to 6-wk-old male mice (nude/athymic mice, NCI) using our brain tumor model as previously described [98]. Mice were sacrificed 8 wk after injection. Brains were fixed using transcardiac perfusion, postfixed overnight at 4 °C in 4% formalin, embedded in OCT compound (Tissue-Tek), and frozen, sectioned, and stained with an antibody against human nestin (1∶500, MAB5326 Millipore). Stained cryosections were used to calculate tumor size and invasiveness by computer-based morphometrics using Image J. Please refer to Text S1 for detailed description of the intracranial injection of GB BTSCs.

### Assessment of Proliferation

Primary human GB cells expressing the control shRNA and NKCC1 shRNA were treated with 10 µM 5-ethynyl-20-deoxyuridine (EdU). Cells were harvested for detection of EdU incorporation using Click-iT EdU Flow Cytometry Assay Kits (Invitrogen, Cat. No. C35002) following the manufacturer's instructions. The percentage of cells that incorporated EdU was measured using flow cytometric detection of EdU. Data were analyzed using Kaluza software (Beckman Coulter).

### Tissue Microarray

A tissue microarray was designed and built according to previously established methods [99]. Cores were taken from each tumor mass or control tissue (see Table S1). The tissue that was included in the cores of the microarray was representative of the tissue blocks from where the cores were obtained. Analysis and correction for cell number was done using the FRIDA software (free web-based tissue microarray analysis software).

### Statistical Analysis

Unless otherwise noted, data are presented as mean ± standard error of the mean. A *t* test was used to compare two groups; one-way analysis of variance (ANOVA) was used in multiple group comparisons with Bonferroni's post hoc test. Mann-Whitney rank-sum test was used to evaluate the statistical significance in quantification of spindle shape factor where indicated. In order to satisfy the distributional assumptions associated with the ANOVA, cell traction force data were first converted to log scale prior to analyses. For the comparisons between treatments, we used a nested ANOVA. All analyses were performed in Sigma Plot 9.0 (Systat Software Inc., San Jose, CA) SAS Version 9.2 (SAS Institute, Cary, NC), and a two-sided *p* value less than 0.05 was considered significant.

## Supporting Information

Figure S1 NKCC1 activity is necessary for GB cell invasion. Quantification of transwell invasion assays of NS 221 exposed to (A) increasing doses of bumetanide. DMSO versus 50 µM. * *p* value<0.05. And (B) NS 561, NS 318, NS 221, NS 319, and NS 243 primary human GB cell lines exposed to 50 µM of bumetanide. (C) Immunoblot showing effective knockdown of NKCC1 in stably transduced NKCC1 shRNA cell lines. (D) Quantification of transwell invasion assays of NS 567 and NS 501 primary human glioma cell lines stably transduced with NKCC1 shRNA. (E) RT-PCR showing stable knockdown of KCC4 in NS 318. Bars represent mean ± S.E.M. * *p* value<0.05. Scale bars represent 50 µm.
(TIF)

Figure S2 NKCC1 knockdown does not decrease proliferation of primary human GB cell lines in vitro or in vivo. (A) Proliferation was measured using Click iT EdU kit (Invitrogen). The fraction of EdU positive cells was similar in cells expressing control shRNA and in cells expressing NKCC1 shRNA in all three cell lines tested: NS 561, NS 501, and NS 318. (B) Quantification of Ki67 positive cells in sections of the in vivo tumors showing no differences in the amount of proliferating cells *n* = 4 mice (12 sections per mouse) for Control shRNA and *n* = 4 mice (12 sections per mouse) for NKCC1 shRNA cells; the differences were not significant. (C) Representative images of Ki67 immunohistochemistries and DAPI of the tumor grafts (top panels, Control shRNA; bottom panels, NKCC1 shRNA). Bar represents 20 µm.
(TIF)

Figure S3 NKCC1 knockdown decreases migration speed and directionality in primary human GB cell lines. (A) Diagram representing a raw nanopatterned surface (left). After ECM-coating (red), cells migrate following the mechanical cues (right). (B) Representative scanning electron microscopy image of the nanopatterned substrate. (C) Representative phase contrast image of GB cells aligned to a nanopatterned surface. (D) Quantification of cell migration on a nanopattern surface when cells are exposed to the NKCC1 inhibitor bumetanide. Bars represent mean ± S.E.M. * *p* value<0.05.
(TIF)

Figure S4 NKCC1 knockdown increases the size of focal adhesions in primary human GB cell lines. NS 501 control shRNA (left panel), NKCC1 shRNA (middle panel), and wild-type treated with 5 µM paclitaxel (right panel) stained with an anti-vinculin antibody (A) and anti-paxillin antibody (B) to visualize focal adhesions.
(TIF)

Figure S5 A detailed description of this technique is given by Butler and colleagues [97],[101]. In brief, cells are plated sparsely on polyacrylamide elastic gel block coated with collagen type I (0.2 µg/ml) and allowed to spread and stabilize for 24 h. (A) Phase contrast image of a single primary human glioma cell adhered to the elastic gel substrate. For each adherent cell, images of fluorescent microbeads (B), 0.2 µm in diameter (Molecular Probes, Eugene, OR), embedded near the gel apical surface are taken at different times; the fluorescent image of the same region of the gel after detachment of the cell with trypsin is used as the reference (traction-free) image. The displacement field between a pair of images is then obtained by identifying the coordinates of the peak of the cross-correlation function [97],[101],[102]. From the displacement field (C) and known elastic properties of the gel, the traction field is calculated using both unconstrained and constrained Fourier transform traction cytometry [97],[101],[102]. The computed traction field is then used to obtain contractile moment, which is a scalar measure of the cell's contractile strength that requires no estimation of cell geometry [97],[101]. Here contractile moment is expressed in pico-Newton meters (pNm).
(TIF)

Figure S6 NKCC1 localizes to the extending processes and colocalizes with WNK3 immunoreactivity in primary human GB cells. (A) Images of NS 319 cells immunostained with T4 antibody (red, left panel), WNK3 antibody (green, center panel), and DAPI (blue). Merge in the right panel showing co-localization of NKCC1 and WNK3 immunoreactivity. (B) NKCC1 localizes to the edge of extending processes in multiple primary human GB cell lines. Scale bars, 50 µm.
(TIF)

Figure S7 Confocal mages of NS 318 cells transfected with NKCC1-GFP migrating on a flat surface at (A) 0 min, (B) 12 min, (C) 20 min, and (D) 36 min. Note localization of NKCC1-GFP in the extending lamellipodia as demonstrated by the arrowheads. Scale bars represent 100 µm.
(TIF)

Figure S8 Assessment of NKCC1 knockdown efficiency of five different shRNA sequences using real-time RT-PCR. Bar chart showing fold change in mRNA levels of NKCC1 in NS 253 glioma cell line expressing five different shRNA sequences. shRNA #2 showed the best knockdown efficiency when compared to control shRNA.
(TIF)

Figure S9 Assessment of differentiation of NS 551 BTSCs into the three neuronal lineages. Immunostains of NS 551 cells differentiated against Tuj1 (neuronal marker), GFAP (astrocytic marker), and NG2 (oligodendroglial marker).
(TIF)

Text S1 Supplemental experimental procedures.
DOC

Video S1 NKCC1-EGFP localizes to extending processes of migrating GB cells on nanopatterned surfaces. NS 318 cells transfected with NKCC1 GFP were plated on a nanopatterned surface, and timelapse imaging was obtained showing that NKCC1-EGFP localizes to the extending processes of migrating GB cells.
(MOV)

Video S2 NKCC1-EGFP localizes to extending processes of migrating GB cells on flat surfaces. NS 318 cells transfected with NKCC1-EGFP were imaged using a spinning disk confocal microscope that allowed us to obtain better spatial resolution of the localization. This experiment shows how NKCC1-EGFP localizes exactly to the membrane of ruffles of extending lamellipodia.
(AVI)

Video S3 Migration of brain tumor cells on a nanopatterned surface. NS 318 cells were plated on a nanopatterned surface that provides cells with nanomechanical cues that attempt to recapitulate the extracellular matrix. Please note how cells migrate in a linear fashion in parallel to the nanogrooves. Nanogrooves are horizontally oriented in this experiment.
(MOV)

## Abbreviations

AA: anaplastic astrocytoma
AE: anion exchangers
AQP4: aquaporin 4
BTSC: brain tumor stem cells
ECM: extracellular matrix
EGF: epidermal growth factor
ERM: ezrin-radixin-moesin
FAK: focal adhesion kinase
FTTM: Fourier Transform Traction Microscopy
GB: Glioblastoma
GEFs: guanine nucleotide exchange factors
NHE1: Na^+^/H^+^ exchanger 1
NKCC1: Na^+^-K^+^-Cl^−^ cotransporter 1
PDK1: phosphoinositide-dependent kinase 1
PI3K: phosphoinositide 3-kinase
TMA: tissue microarray
WM: wortmannin
WNK: With No K-lysine
