# Supplementary figures and images for: Regulation of Brain Tumor Dispersal by NKCC1 Through a Novel Role in Focal Adhesion Regulation
Source: PLoS Biol. 2012 May 1;10(5):e1001320. doi: 10.1371/journal.pbio.1001320 (PMC3341330; doi:10.1371/journal.pbio.1001320)

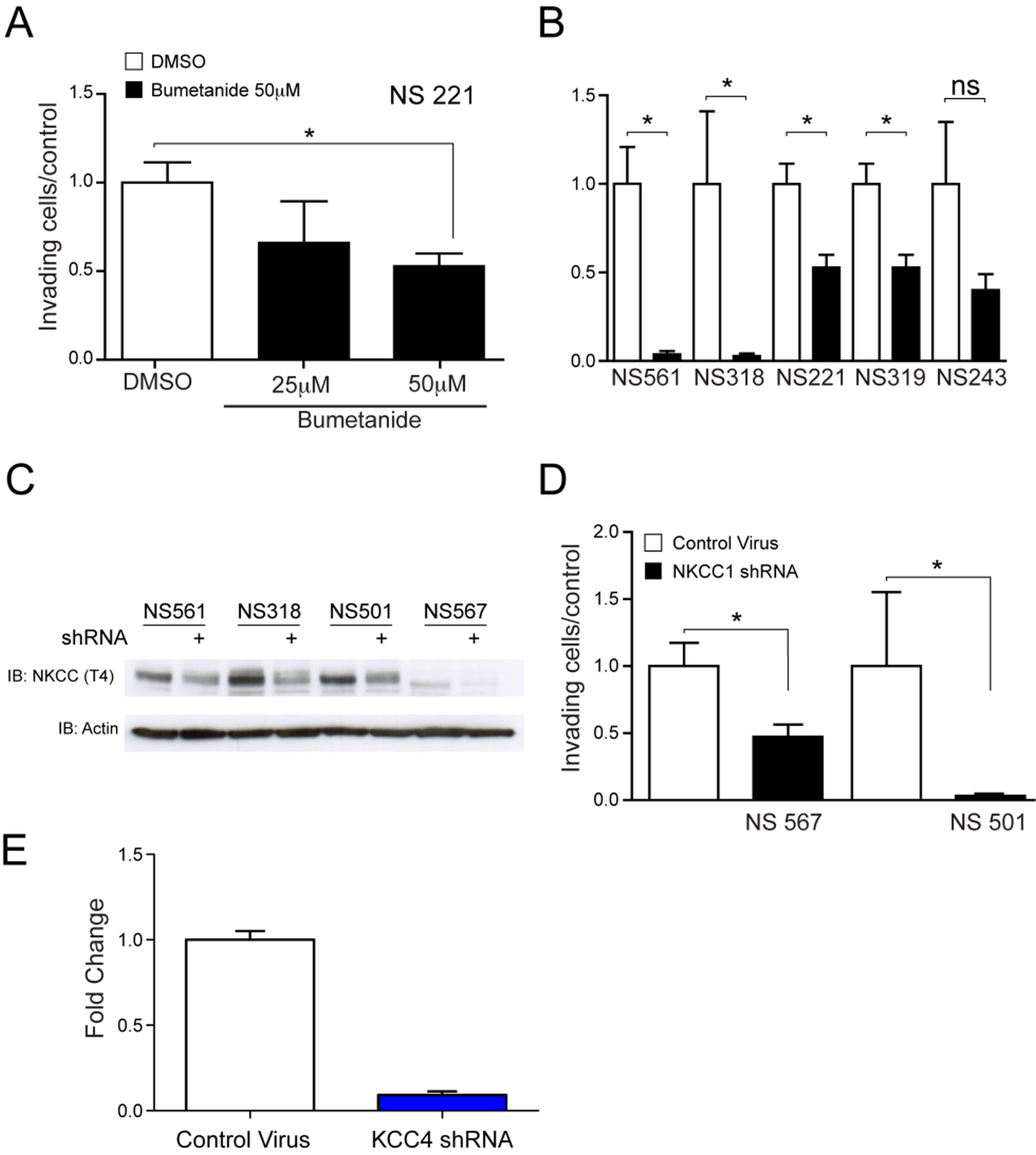

Supplement: Figure S1 — NKCC1 activity is necessary for GB cell invasion. Quantification of transwell invasion assays of NS 221 exposed to (A) increasing doses of bumetanide. DMSO versus 50 µM. * p value<0.05. And (B) NS 561, NS 318, NS 221, NS 319, and NS 243 primary human GB cell lines exposed to 50 µM of bumetanide. (C) Immunoblot showing effective knockdown of NKCC1 in stably transduced NKCC1 shRNA cell lines. (D) Quantification of transwell invasion assays of NS 567 and NS 501 primary human glioma cell lines stably transduced with NKCC1 shRNA. (E) RT-PCR showing stable knockdown of KCC4 in NS 318. Bars represent mean ± S.E.M. * p value<0.05. Scale bars represent 50 µm. (TIF) [file pbio.1001320.s001.tif]

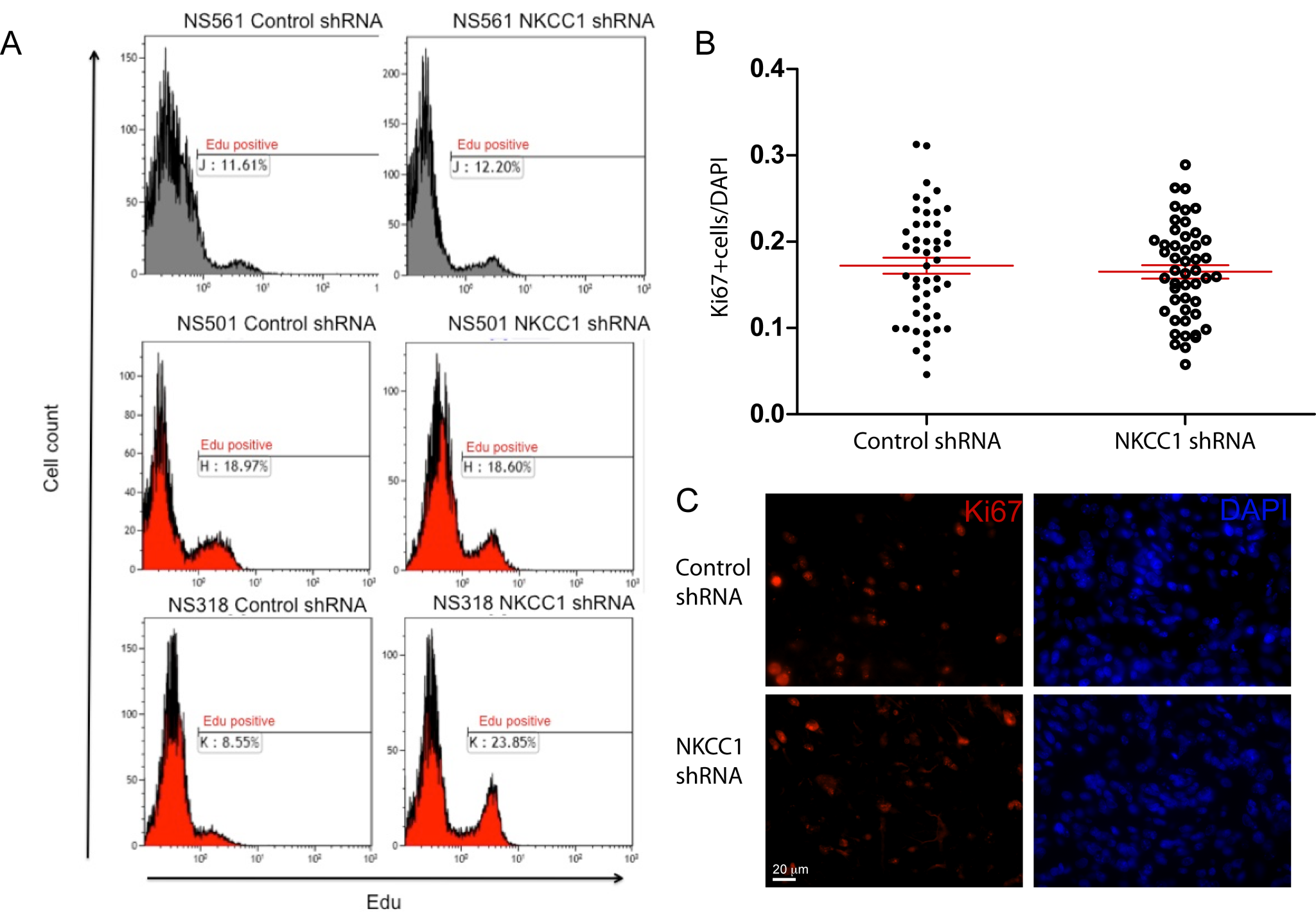

Supplement: Figure S2 — NKCC1 knockdown does not decrease proliferation of primary human GB cell lines in vitro or in vivo. (A) Proliferation was measured using Click iT EdU kit (Invitrogen). The fraction of EdU positive cells was similar in cells expressing control shRNA and in cells expressing NKCC1 shRNA in all three cell lines tested: NS 561, NS 501, and NS 318. (B) Quantification of Ki67 positive cells in sections of the in vivo tumors showing no differences in the amount of proliferating cells n = 4 mice (12 sections per mouse) for Control shRNA and n = 4 mice (12 sections per mouse) for NKCC1 shRNA cells; the differences were not significant. (C) Representative images of Ki67 immunohistochemistries and DAPI of the tumor grafts (top panels, Control shRNA; bottom panels, NKCC1 shRNA). Bar represents 20 µm. (TIF) [file pbio.1001320.s002.tif]

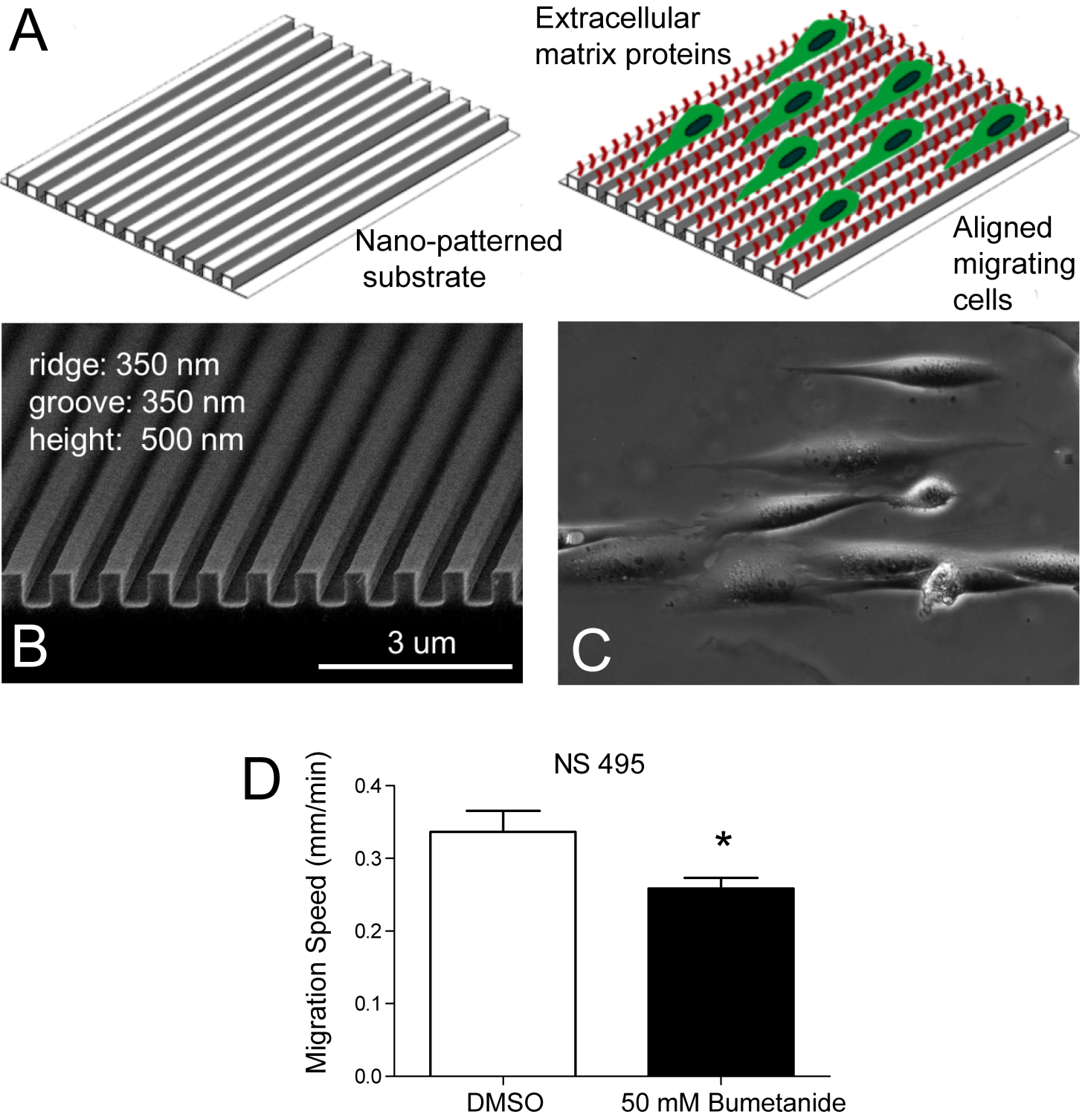

Supplement: Figure S3 — NKCC1 knockdown decreases migration speed and directionality in primary human GB cell lines. (A) Diagram representing a raw nanopatterned surface (left). After ECM-coating (red), cells migrate following the mechanical cues (right). (B) Representative scanning electron microscopy image of the nanopatterned substrate. (C) Representative phase contrast image of GB cells aligned to a nanopatterned surface. (D) Quantification of cell migration on a nanopattern surface when cells are exposed to the NKCC1 inhibitor bumetanide. Bars represent mean ± S.E.M. * p value<0.05. (TIF) [file pbio.1001320.s003.tif]

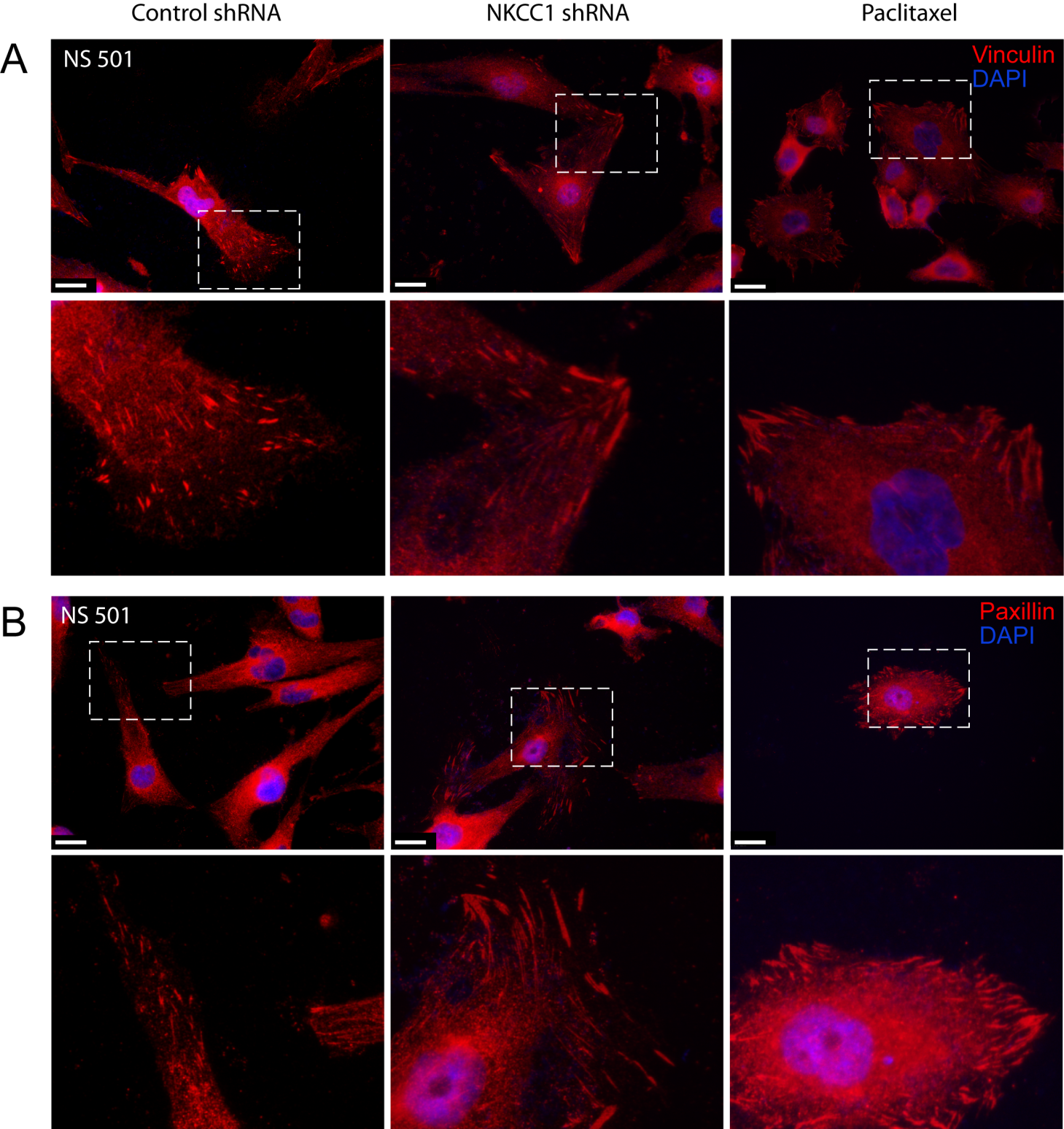

Supplement: Figure S4 — NKCC1 knockdown increases the size of focal adhesions in primary human GB cell lines. NS 501 control shRNA (left panel), NKCC1 shRNA (middle panel), and wild-type treated with 5 µM paclitaxel (right panel) stained with an anti-vinculin antibody (A) and anti-paxillin antibody (B) to visualize focal adhesions. (TIF) [file pbio.1001320.s004.tif]

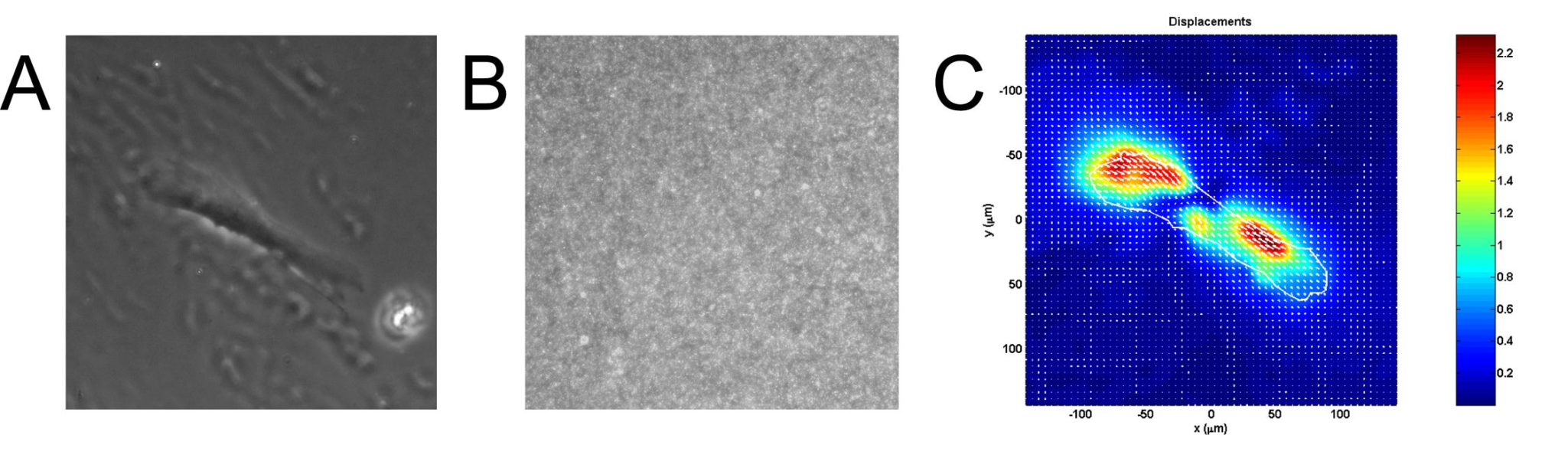

Supplement: Figure S5 — A detailed description of this technique is given by Butler and colleagues [97],[101]. In brief, cells are plated sparsely on polyacrylamide elastic gel block coated with collagen type I (0.2 µg/ml) and allowed to spread and stabilize for 24 h. (A) Phase contrast image of a single primary human glioma cell adhered to the elastic gel substrate. For each adherent cell, images of fluorescent microbeads (B), 0.2 µm in diameter (Molecular Probes, Eugene, OR), embedded near the gel apical surface are taken at different times; the fluorescent image of the same region of the gel after detachment of the cell with trypsin is used as the reference (traction-free) image. The displacement field between a pair of images is then obtained by identifying the coordinates of the peak of the cross-correlation function [97],[101],[102]. From the displacement field (C) and known elastic properties of the gel, the traction field is calculated using both unconstrained and constrained Fourier transform traction cytometry [97],[101],[102]. The computed traction field is then used to obtain contractile moment, which is a scalar measure of the cell's contractile strength that requires no estimation of cell geometry [97],[101]. Here contractile moment is expressed in pico-Newton meters (pNm). (TIF) [file pbio.1001320.s005.tif]

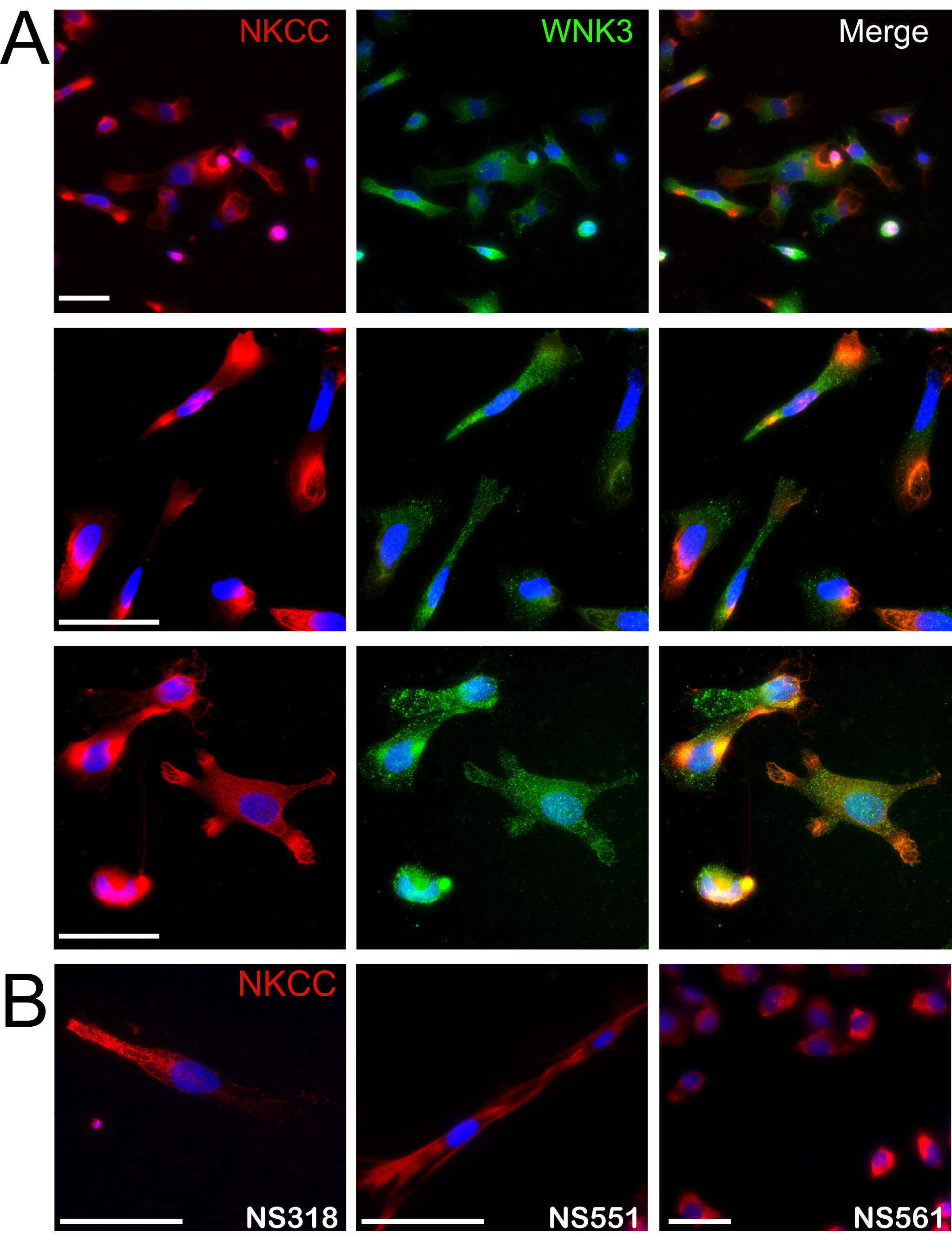

Supplement: Figure S6 — NKCC1 localizes to the extending processes and colocalizes with WNK3 immunoreactivity in primary human GB cells. (A) Images of NS 319 cells immunostained with T4 antibody (red, left panel), WNK3 antibody (green, center panel), and DAPI (blue). Merge in the right panel showing co-localization of NKCC1 and WNK3 immunoreactivity. (B) NKCC1 localizes to the edge of extending processes in multiple primary human GB cell lines. Scale bars, 50 µm. (TIF) [file pbio.1001320.s006.tif]

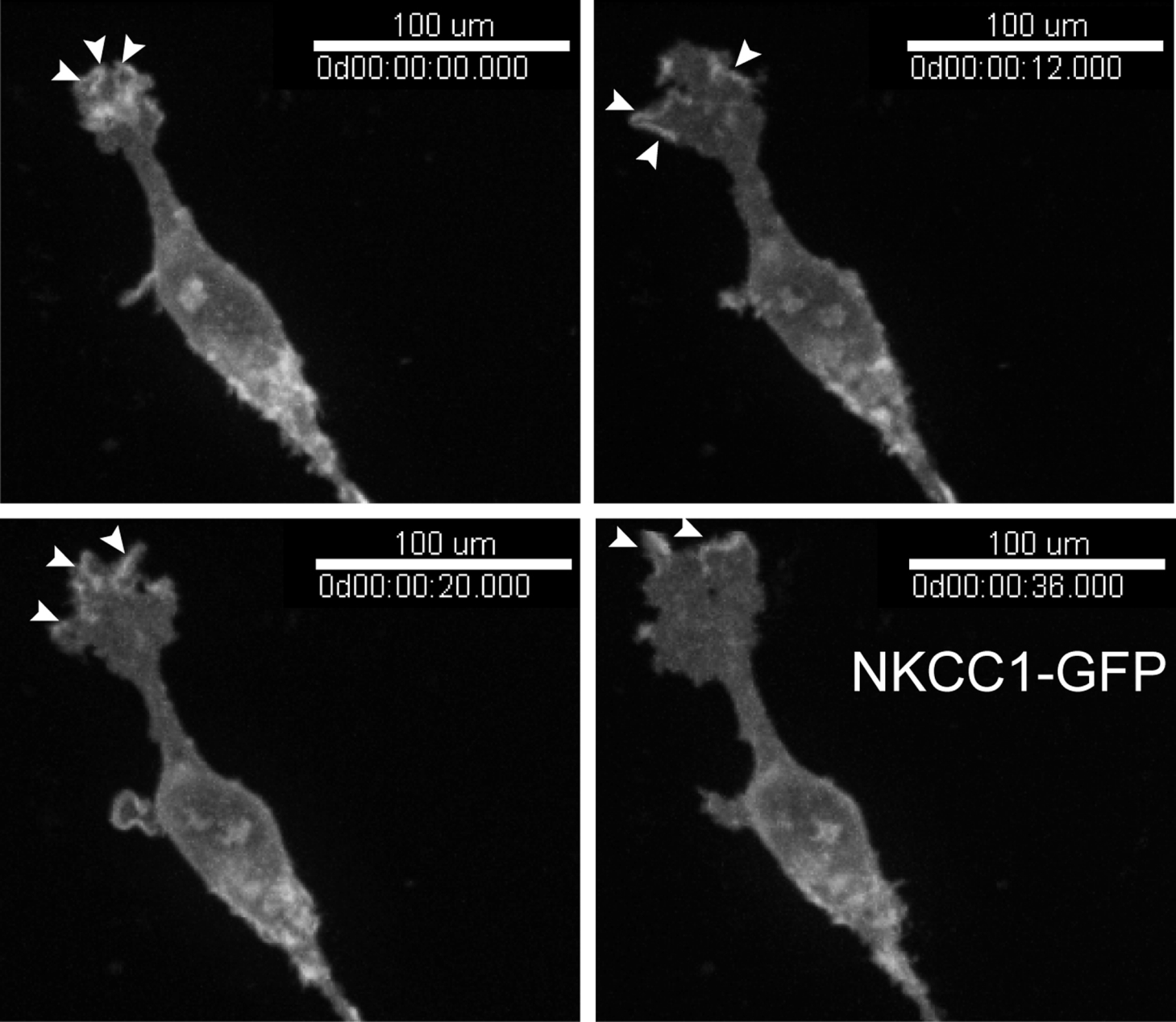

Supplement: Figure S7 — Confocal mages of NS 318 cells transfected with NKCC1-GFP migrating on a flat surface at (A) 0 min, (B) 12 min, (C) 20 min, and (D) 36 min. Note localization of NKCC1-GFP in the extending lamellipodia as demonstrated by the arrowheads. Scale bars represent 100 µm. (TIF) [file pbio.1001320.s007.tif]

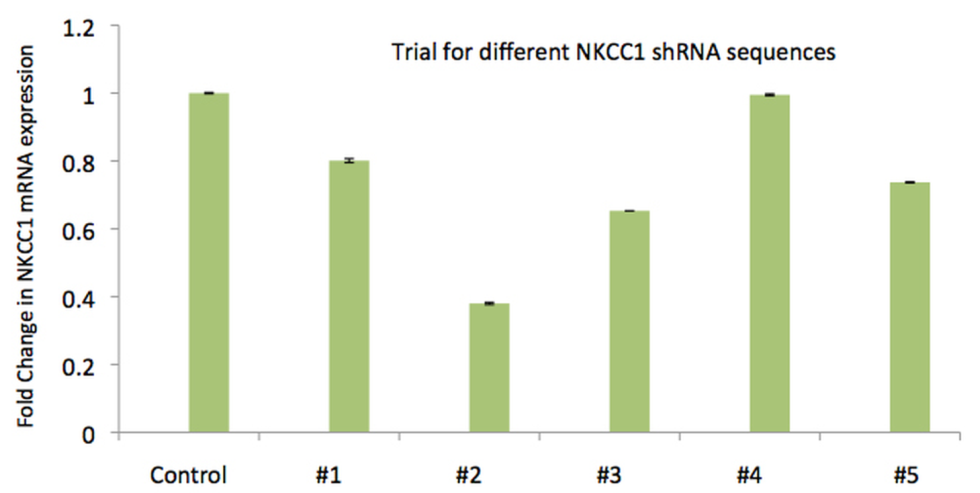

Supplement: Figure S8 — Assessment of NKCC1 knockdown efficiency of five different shRNA sequences using real-time RT-PCR. Bar chart showing fold change in mRNA levels of NKCC1 in NS 253 glioma cell line expressing five different shRNA sequences. shRNA #2 showed the best knockdown efficiency when compared to control shRNA. (TIF) [file pbio.1001320.s008.tif]

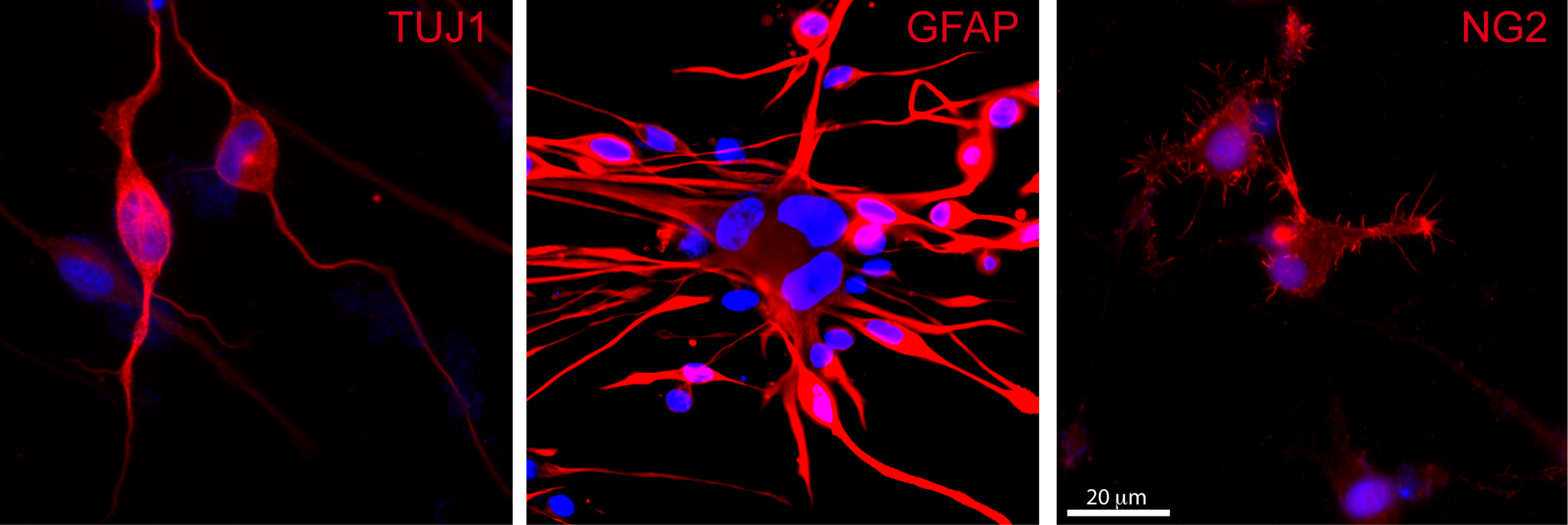

Supplement: Figure S9 — Assessment of differentiation of NS 551 BTSCs into the three neuronal lineages. Immunostains of NS 551 cells differentiated against Tuj1 (neuronal marker), GFAP (astrocytic marker), and NG2 (oligodendroglial marker). (TIF) [file pbio.1001320.s009.tif]
