# Supplementary material for: Regulation of Brain Tumor Dispersal by NKCC1 Through a Novel Role in Focal Adhesion Regulation
Source: PLoS Biol. 2012 May 1;10(5):e1001320. doi: 10.1371/journal.pbio.1001320 (PMC3341330; doi:10.1371/journal.pbio.1001320)
Supplement: Text S1 — Supplemental experimental procedures. DOC [file pbio.1001320.s010.doc]

**Supplemental Experimental Procedures:**

***Cell lines***. Patient samples of glioma tissues were obtained at the Johns Hopkins Hospital under the approval of the Institutional Review Board (IRB). All human brain tumor cell lines were derived from intraoperative tissue samples from patients treated surgically for newly diagnosed glioblastoma multiforme without prior treatment as listed in **Table S2**. Primary GB cells were cultured in Dulbecco's Modified Eagle Medium: Nutrient Mixture F-12 in 10% fetal bovine serum (Invitrogen). Primary brain tumor stem cell (BTSC) lines were cultured as neurospheres in media consisting of Dulbecco's Modified Eagle Medium: Nutrient Mixture F-12, B27 serum free supplement (Gibco), 20ng/mL epidermal growth factor (EGF), and 20ng/ml fibroblast-derived growth factor (FGF). The BTSC line 551 was shown to self renew (as it was passaged more than 8 times) and be tumorigenic (**Figure 1**), and differentiate into different neuronal lineages (**Figure S9**). Detailed culture methodology has been previously described . HEK293 (Human Embryonic Kidney) cells for phosphorylation analysis were obtained from ATCC (American Type Culture Collection, Manassas, VA, USA) and cultured according to manufacturer instructions.

***Generation of NKCC1-shRNA stable expressing cell lines****.* A Mission human clone set of sequence verified plasmids (pLKO.1) that target human NKCC1 (SLC12A2). Five different shRNA sequences directed against NKCC1 obtained from the “Sigma Mission shRNA” were tested in one cell line. Among these five different shRNA sequences, the one that achieved the highest silencing of NKCC1 transcript was shRNA number 2, which was chosen to generate the stable cell lines (**Figure S8**). VSV-G pseudotyped virus was produced by co-transfecting 293T cells with a shRNA transducing vector and two packaging vectors: psPAX2 and pMD2.G. Based on quantitative real-time PCR (RT-PCR) experiments using the primer sense 5’-CAA GAC ATA CCG GCA GAT CAG-3’ and antisense 5’-ACT AGA CAC AGC ACC TTT TCG-3’ it was determined that construct number 2 (with sequence 5’-TAG TGC TCT CTA CAT GGC ATG GTT AGA AGC TCT ATC TAA GGA CCT ACC ACC AAT CCT C-3’) provided the best knockdown efficiency and was used to transduce primary glioma cell lines NS318, NS561, NS501, NS567 (grown in 10% fetal bovine serum), and BTSC551 (grown as neurospheres). Seventy-two hours after transduction, cells were cultured in the presence of puromycin for selection of cells expressing the shRNA. Knockdown was assessed by immunoblot (**Figure 1F and Figure S1C**) or by RT-PCR (**Figure S1E**).

***Cloning of full-length human NKCC1 cDNA, site directed mutagenesis, subcloning into lentiviral vector, and generation of EGFP fusion protein****.* In order to clone the full-length open reading frame of NKCC1 (SLC12A2, accession number NM001046), total RNA was extracted from an astrocytic human cell line. The cell line was derived from a tissue sample of the area surrounding the lateral ventricles in a patient with epilepsy using the RNeasy RNA isolation kit (Qiagen). cDNA was synthesized with the SuperScript™ III First-Strand Synthesis System with random hexamers as indicated in the manufacturer’s instructions (Invitrogen). Gene-specific primers were designed in order to amplify the full-length of the open reading frame of NKCC1 by polymerase chain reaction (PCR) using high fidelity PFU fusion polymerase (Agilent Technologies). The sequence of the primers employed is as follows: sense, 5’- GCG TGC TGC CGG AGA CGT CC-3’; antisense, 5’- AGT CAC CAT TCG CCA TTG TGA TGT T-3’. The resulting PCR product of ~3,890 base pairs, which included the whole open reading frame, was cloned into pCR-XL-TOPO (Invitrogen). The cloned sequence was verified in its entirety to confirm the absence of mutations. In order to make a NKCC1-EGFP fusion protein it was necessary to insert a XhoI site in the 3’ end of the open reading frame 4 bases before the stop codon with the following primers: sense 5’- GTC CTT ACC TTC TAT TCt cgA gTG TTC TAT ACA GTG GAC AG-3’ and antisense 5’- CTG TCC ACT GTA TAG AAC AcT cga GAA TAG AAG GTA AGG AC-3’. Subsequently, NKCC1 was subcloned into pcDNA3-EGFP as a EcoRI-XhoI fragment. The changes are noted in the lowercase letters and were introduced using the Quikchange-XL site directed mutagenesis kit (Agilent Technologies, Cat. No. 200521) according to manufacturer’s instructions. Site-directed mutagenesis was also confirmed by sequencing.

***Quantitative real-time polymerase chain reaction****.* Total RNA was extracted from primary glioma cell lines using the RNAeasy kit (Qiagen). Approximately 1 µg of total RNA for each sample was primed using oligo-dT and reverse transcribed using the SuperScript™ III First-Strand Synthesis System for RT-PCR (Invitrogen) following manufacturers instructions. The target transcript was amplified using specific primers (mentioned above) and using SYBR Green PCR master mix (Applied Biosystems) in a 7300 Real-Time PCR system (Applied Biosystems). For relative quantification, the results obtained were compared to the levels of target mRNA expression present in the control cell line and normalized for GAPDH expression.

***Immunoblotting****.* Cells were plated on a 10 cm dish and exposed to the different experimental conditions. At the end of the experiment, cell were harvested using a cell scraper in RIPA lysis buffer (150mM NaCl, 10mM Tris, pH 7.5, 1% NP40, 1% deoxycholate, 0.1% SDS, protease inhibitor cocktail (Roche), and Halt^TM^ Phosphatase inhibitor coctail (Thermo)). Proteins from whole cell lysates were resolved using the NuPAGE 4–12% Bis-Tris gradient gel (Invitrogen). Proteins were transferred to PVDF membrane, blocked in 5% non-fat milk or 2% bovine serum albumin in TBS-Tween-20, and probed with the antibodies for NKCC1 (T4, 1:500, Developmental Studies Hybridoma Bank, University of Iowa), phospho-NKCC1 (R5, 1:10,000, kindly provided by Dr. Biff Forbush, Yale University), beta-actin (1:10,000, Abcam), Akt (1:1000, Cell Signaling), phospho-Akt (1:1000, Cell Signaling), and WNK3 (1:2500, Bethyl Laboratories). Phospho-(Ser/Thr) Akt Substrate Antibody that recognizes phosphorylated (R/K)X(R/K)XX(T*/S*) motifs (1:2500, Cell Signaling) was also employed. Detection was done with the appropriate horseradish-peroxidase conjugated secondary antibodies and using the enhanced chemiluminescence reagent (GE Healthcare Life Sciences).

***Immunoprecipitation****.* The immunoprecipitation lysis buffer was 0.25M NaCl, 50mM Tris pH 8, 0.5 mM EDTA, pH 8, 0.1% (by mass) NP-40. Protease inhibitor cocktail (Roche) and Halt^TM^ Phosphatase inhibitor cocktail (Thermo) were added to the lysis buffer. Cell lysates (150μg of protein) were incubated with anti NKCC antibody (T4 antibody, 1μg; Developmental Hy) overnight at 4°C on a shaking platform. Subsequently, 50μL of protein G magnetic beads (Millipore) were added to the protein-antibody mix and incubated at room temperature for 10 minutes. Beads were precipitated magnetically and washed three times in PBS-0.1% Tween. Proteins were then eluted and denatured in LDS protein loading buffer (Invitrogen).

***In vitro* *invasion assay***. For transwell invasion assays 50,000 cells were plated in the top chamber with a matrigel-coated membrane (24-well insert; pore size, 8 mm; BD Biosciences). Cells were plated in medium containing 0.5% of serum whereas medium with 2% serum was used as a chemo-attractant in the lower chamber. Cells were incubated for 48 h and cells that did not invade through the pores were removed thoroughly using a cotton swab. Cells on the lower surface of the membrane were stained using the Diff-Quick Staining Set (Dade) and cells of 9 pre-established high power fields in the membrane were counted. Bumetanide (Sigma-Aldrich), a specific NKCC inhibitor, and DIOA (R(+)-Butylindazone, R-(+)-[(2-*n*-Butyl-6,7-dichloro-2-cyclopentyl-2,3-dihydro-1-oxo-1H-inden-5-yl)oxy]acetic acid, Sigma-Aldrich) a potent inhibitor of KCC transport were used in pharmacological inhibition experiments. Both pharmacological agents were dissolved in DMSO, which was also applied to the controls of the experiments.

***Nanogrooved pattern cell migration assay***. Migration of glioma cells was quantified using a novel directional migration assay using nano-ridges/grooves of 350 nm in groove width, 350 nm in ridge width, and 500 nm in depth, constructed of transparent poly(urethane acrylate) (PUA), and fabricated using UV-assisted capillary lithography (See **Figure S3)**. This nanopatterned subrstratum attempts at reproducing the extracellular matrix in real tissue, giving mechanical directional cues to attached cells. Cells were cultured on glass covered with a nanogrooved pattern substratum, which was previously glued onto the bottom surface of the custom-made Mat-tek dish (P35G-20-C). Prior to plating cells, nanogrooved substrata were coated with poly-D-lysine (10 ug/ml concentration) for 15 minutes and laminin (10 ug/ml concentration) for 1 hour. These topographically patterned cell substrata, caused cells to align with and move along the direction of the nanogrooves. Cell migration was quantified using timelapse microscopy (**Movie S3**). To enable long-term observation, a custom-modified Mat-tek dish, integrated with the topographically patterned substratum, was mounted onto the stage of a motorized inverted microscope (Olympus IX81) equipped with a Cascade 512B II CCD camera and temperature and gas controlling environmental chamber. Phase-contrast and epi-fluorescent cell images were automatically recorded under 10X objective (NA = 0.30) using the Slidebook 4.1 (Intelligent Imaging Innovations, Denver, CO) for 15 hours at 10-20 minute intervals. Cell migration assays and their particular features are summarized in **table S3**.

***Quantitative analysis of cell migration***. A custom-made MATLAB script was used to identify cell boundaries from phase-contrasted images and to measure cell centroid positions. Average individual cell speeds (S) were calculated from individual cell trajectories and durations of the image acquisition. Mean squared displacements (MSD) at various time intervals (t) were calculated using the method of non-overlapping intervals and directional persistence time (P) was obtained by fitting them to the persistent random walk model :

*MSD* = 2*S*^2^*P*[*t* – *P*(1 – *e* –*^t^*/*^P^*)]

Because cell-cell contact is known to affect the extent of cell spreading and migration, cells were plated at low density allowing isolated movements. The axial ratio was defined as the ratio of the maximal cell cross-section length along the nanogrooves to that across the grooves (longitudinal vs. transverse directions). The spindle shape factor was defined as the ratio of the length of maximum cell width (maximal axis) to the minimum value of the cell width in the direction perpendicular to maximum axis, regardless of the orientation with respect to nanogrooves. For each condition, over 60 cells were quantified in total.

For quantitative analysis of cell orientation, we cultured cells on the topographically defined substratum for 23 h. Cells were fixed and stained for F-actin with phalloidin. The orientation angle of polarized cell was determined by measuring the acute angle between the major axis of the cell and the direction of grooves. More than 100 cells for each group were used to construct the polarization angle distributions with range −90° and 90°. Positive and negative angles were defined to be counter-clockwise and clockwise direction, respectively. An angle of 0° was defined as the angle when cells were perfectly aligned parallel to the ridge/groove pattern arrays.

***Fourier transform traction microscopy (FTTM)****.* The contractile stress arising at the interface between an adherent cell and its substratum was measured with traction microscopy .  Cells were plated sparsely on elastic gel blocks (Young’s modulus of 8 kPa with the Poisson’s ratio of 0.48), and allowed to adhere and stabilize for 24 h.  For each cell analyzed, the traction field was computed using Fourier transform traction cytometry as described previously . The computed traction field was used to obtain the net contractile moment, which is a scalar measure of the cell’s contractile strength (**Figure S5**).  Net contractile moment was expressed in the units of pico-Newton- meters (pNm).

***Immunofluorescence****.* GB cells were plated on 15mm glass coverslips (Warner Instruments) coated with laminin (5 μg /cm^2^ diluted in serum free DMEM-F12 culture media) and Poly-D lysine (10 μg/μl). Cells were fixed in 4% paraformaldehyde (pH7.4) for one hour, washed 3 times, five minutes per wash with phosphate-buffered saline (PBS, pH7.4), and blocked with 10% normal donkey serum in PBS for 1 hour. Subsequently, fixed cells were incubated with primary antibody at 4°C overnight. Primary antibody was aspirated and washed three times with PBS and the preparation was incubated with Alexa Fluor-conjugated secondary antibodies (1:500, Invitrogen) and mounted using Aquamount (VWR). The cells were imaged using an Olympus IX81 epifluorescence microscope. Paxillin and vinculin positive areas were detected automatically and the area was calculated using IMARIS (Bitplane, Scientific Software).

***Intracranial GB cell injections, sectioning, and histochemistry***. All animal protocols were approved by the Johns Hopkins Animal Care and Use Committee. In vivo invasion and tumorigenesis of cells expressing NKCC1 shRNA were assessed in 4- to 6-wk-old male mice (nude/athymic mice, NCI) using our brain tumor model as previously described . Cells in 1μL of serum free media (100,000 cells/ μL) were injected in the right hemisphere in the corpus callosum overlying the striatum of athymic nude mice (NCI) under stereotactic control (a bur hole was placed at coordinates X=1.5 mm and Y=1.34 mm relative to the bregma and cells were injected at a depth of -2.5 mm). Mice were sacrificed 8 weeks after injection. Brains were fixed using transcardiac perfusion, postfixed overnight at 4°C in 4% formalin, cryopreserved in 30% sucrose, embedded in OCT compound (Tissue-Tek) and frozen, and sectioned and stained with an antibody against human nestin (1:500, Millipore). Stained cryosections were used to calculate tumor size and invasiveness by computer-based morphometrics using Image J.

***Assessment of proliferation****.* Primary human GB cells expressing the control shRNA and NKCC1 shRNA were treated with 10 μM 5-ethynyl-20-deoxyuridine (EdU), a thymidine analogue that is incorporated to DNA during the S-phase of cell cycle for 18 hours prior to the assessment of proliferation. Cells were harvested and subjected to aldehyde-based fixation and permeabilization for detection of EdU incorporation using Click-iT™ EdU Flow Cytometry Assay Kits (Invitrogen, Cat. No. C35002) following manufacturers instructions. The percentage of cells that incorporated EdU was measured using flow cytometric detection of EdU. Data was analyzed using Kaluza software (Beckman Coulter).

#### *Tissue microarray*. A tissue microarray was designed and built according to previously established methods . Cores were taken from each tumor mass or control tissue. NKCC1 levels were evaluated by immunohistochemistry using a NKCC1 specific antibody on over 80 unique glioma and normal tissue samples. Slides were digitally scanned, and quantitative analysis was performed using a color deconvolution image analysis technique as explained previously by our group . The array of tissue contained samples from 20 GB, 21 anaplastic astrocytomas (AA), 19 low-grade astrocytomas (A), 3 WHO grade III oligodendrogliomas (O3), 3 oligoastrocytomas (OA), and 2 mixed oligoastrocytomas (OA) (See Table S1). The tissue that was included in the cores of the microarray was representative of the tissue blocks from where the cores were obtained. Slides stained for NKCC1 and counterstained with hematoxylin to visualize the nuclei were scanned using a ScanScope CS (Aperio; Vista, CA). Staining for NKCC1 was analyzed using color deconvolution techniques described previously . A DAB staining threshold value was selected empirically to distinguish between background from true staining. The values obtained were then corrected for the number of nuclei in each slide, which were counted using the same method of color deconvolution. The correction for the cellularity was done using the FRIDA software (free webbased tissue microarray analysis software), developed by the Tissue Microarray Facility at Johns Hopkins University.

***Statistical Analysis***. Unless otherwise noted, data are presented as mean ± standard error of the mean. T -test was used to compare two groups; One-way Analysis of Variance (ANOVA) was used in multiple group comparisons with Bonferroni’s post-hoc test. Mann-Whitney rank-sum test was used to evaluate the statistical significance in quantification of spindle shape factor where indicated. In order to satisfy the distributional assumptions associated with the ANOVA, cell traction force data were first converted to log scale prior to analyses. For the comparisons between treatments, we used a nested ANOVA. All analyses were performed in Sigma Plot 9.0 (Systat Software Inc., San Jose, CA) SAS Version 9.2 (SAS Institute, Cary, NC), and a 2-sided P value less than 0.05 was considered significant.

Table S1. Summary of tissue samples in the tissue microarray

| **Pathological Diagnosis** | **Number of Samples** | **Mean age ± SEM (Years)** | | **Female percentage** |
| --- | --- | --- | --- | --- |
| Glioblastoma (GB) | 20 | 60.5 | 2.6 | 55.0% |
| Anaplastic astrocytoma (AA) | 21 | 37.8 | 2.4 | 42.9% |
| Low-grade astrocytoma (A) | 19 | 37.4 | 2.1 | 42.1% |

Table S2. Summary of cell lines for RNA extraction

|  |  |  |  |
| --- | --- | --- | --- |
| **Cell line** | **Diagnosis** | **Age at resection (years)** | **Gender** |
| NS221 | GLIOBLASTOMA | 71 | Female |
| NS243 | ASTROCYTOMA GRADE II | 45 | Male |
| NS253 | GLIOBLASTOMA | 53 | Female |
| NS318 | GLIOBLASTOMA | 66 | Male |
| NS319 | GLIOBLASTOMA WITH PNET COMPONENT | 74 | Male |
| NS495 | GLIOBLASTOMA | 50 | Male |
| NS501 | GLIOBLASTOMA | 52 | Male |
| NS561 | GLIOBLASTOMA | 55 | Male |
| NS567 | GLIOBLASTOMA | 60 | Male |
| BTSC 551 | GLIOBLASTOMA | 69 | Male |

Table S3. Summary of cell migration assays

| **Type of Assay** | **Characteristics** | **Application** | **Experimental system** | **Result** |
| --- | --- | --- | --- | --- |
| Migration on Nanopatterned Surface | Novel directional migration assay using patterned nanogrooves that allows for optimal quantification of cell migration. | Useful for quantitative study of cell migration parameters such as speed, distance, and direction, as well as morphological characteristics of plated cells. | Nano-ridges/grooves constructed of transparent poly(urethane acrylate) (PUA), coated with laminin. | -NKCC1 pharmacological and genetic inhibition reduced **speed** and migration **directionality.** |
| Transwell migration assay | Standard method to evaluate cell invasion. Based on two chambers that are separated by a filter through which cells migrate. Chemotactic gradients can be set up and cell migration can be quantified. Cell proliferation, possible confounding factor. | Initial evaluation of changes in BTSC migration. | Boyden chamber matrigel-coated membrane (24-well insert; pore size, 8 mm; BD Biosciences) | -NKCC1 pharmacological and genetic inhibition reduced **migration.** |
| Traction force microscopy | Adherent cells exert tractions on their surroundings. These tractions can be measured by observing the displacements of beads embedded on a flexible gel substrate on which the cells are cultured. | Used to measure the contractile stress arising at the interface between an adherent cell and its substratum. | Polyacrylamide elastic gel block coated with collagen type I. Fluorescent microbeads are embedded near the gel apical surface | -NKCC1 KD reduces cell **contractility** (net contractile moments). |

**References:**

1. Butler JP, Tolic-Norrelykke IM, Fabry B, Fredberg JJ (2002) Traction fields, moments, and strain energy that cells exert on their surroundings. Am J Physiol Cell Physiol 282: C595-605.

2. Tolic-Norrelykke IM, Butler JP, Chen J, Wang N (2002) Spatial and temporal traction response in human airway smooth muscle cells. Am J Physiol Cell Physiol 283: C1254-1266.

3. Wang N, Tolic-Norrelykke IM, Chen J, Mijailovich SM, Butler JP, et al. (2002) Cell prestress. I. Stiffness and prestress are closely associated in adherent contractile cells. Am J Physiol Cell Physiol 282: C606-616.

4. Chaichana KL, Guerrero-Cazares H, Capilla-Gonzalez V, Zamora-Berridi G, Achanta P, et al. (2009) Intra-operatively obtained human tissue: protocols and techniques for the study of neural stem cells. J Neurosci Methods 180: 116-125.

5. Guerrero-Cazares H, Chaichana KL, Quinones-Hinojosa A (2009) Neurosphere culture and human organotypic model to evaluate brain tumor stem cells. Methods Mol Biol 568: 73-83.

6. Kim DH, Lipke EA, Kim P, Cheong R, Thompson S, et al. (2010) Nanoscale cues regulate the structure and function of macroscopic cardiac tissue constructs. Proc Natl Acad Sci U S A 107: 565-570.

7. Dickinson R, Tranquillo R (1993) Optimal estimation of cell-movement indexes from the statistical-analysis of cell tracking data. Aiche J 39: 1995-2010.

8. Munevar S, Wang Y, Dembo M (2001) Traction force microscopy of migrating normal and H-ras transformed 3T3 fibroblasts. Biophys J 80: 1744-1757.

9. Guerrero-Cazares H, Attenello F, Noiman L, Quiñones-Hinojosa A (2011 In Press) Stem Cells in Gliomas. In: D G, R S, editors. Handbook of Clinical Neurology: Neuro-oncology Volume. Edinburgh, UK: Elsevier.

10. Kononen J, Bubendorf L, Kallioniemi A, Barlund M, Schraml P, et al. (1998) Tissue microarrays for high-throughput molecular profiling of tumor specimens. Nat Med 4: 844-847.

11. Halushka MK, Selvin E, Lu J, Macgregor AM, Cornish TC (2009) Use of human vascular tissue microarrays for measurement of advanced glycation endproducts. J Histochem Cytochem 57: 559-566.

12. Cornish TC, Halushka MK (2009) Color deconvolution for the analysis of tissue microarrays. Anal Quant Cytol Histol 31: 304-312.
